# Divergent semantic integration (DSI): Extracting creativity from narratives with distributional semantic modeling

**DOI:** 10.3758/s13428-022-01986-2

**Published:** 2022-10-17

**Authors:** Dan R. Johnson, James C. Kaufman, Brendan S. Baker, John D. Patterson, Baptiste Barbot, Adam E. Green, Janet van Hell, Evan Kennedy, Grace F. Sullivan, Christa L. Taylor, Thomas Ward, Roger E. Beaty

**Affiliations:** 1 Department of Cognitive and Behavioral Science, Washington and Lee University, Parmly Hall, 204 W. Washington St, Lexington, VA 24450 USA; 2 Neag School of Education, University of Connecticut, Mansfield, CT USA; 3 Department of Psychology, Pennsylvania State University, State College, PA USA; 4 Psychological Sciences Research Institute, Université catholique de Louvain, Ottignies-Louvain-la-Neuve, Belgium; 5 Department of Psychology, Georgetown University, Washington, DC USA; 6 Department of Psychology, Graceland University, Lamoni, IA USA; 7 Department of Psychology, University of Alabama, Tuscaloosa, AL USA

**Keywords:** Creativity in writing, Narratives, Distributional semantic modeling, Natural language processing, Creativity assessment

## Abstract

We developed a novel conceptualization of one component of creativity in narratives by integrating creativity theory and distributional semantics theory. We termed the new construct *divergent semantic integration* (*DSI*), defined as the extent to which a narrative connects divergent ideas. Across nine studies, 27 different narrative prompts, and over 3500 short narratives, we compared six models of *DSI* that varied in their computational architecture. The best-performing model employed Bidirectional Encoder Representations from Transformers (BERT), which generates context-dependent numerical representations of words (i.e., embeddings). BERT *DSI* scores demonstrated impressive predictive power, explaining up to 72% of the variance in human creativity ratings, even approaching human inter-rater reliability for some tasks. BERT *DSI* scores showed equivalently high predictive power for expert and nonexpert human ratings of creativity in narratives. Critically, *DSI* scores generalized across ethnicity and English language proficiency, including individuals identifying as Hispanic and L2 English speakers. The integration of creativity and distributional semantics theory has substantial potential to generate novel hypotheses about creativity and novel operationalizations of its underlying processes and components. To facilitate new discoveries across diverse disciplines, we provide a tutorial with code (osf.io/ath2s) on how to compute *DSI* and a web app (osf.io/ath2s) to freely retrieve *DSI* scores.

Is identifying creativity in writing ineffable in nature, leaving it out of science’s reach (Carey, 2006)? Or are there defining characteristics that reliably distinguish highly creative texts? There is mounting evidence that humans can agree on some of creativity’s key components in narratives (Barbot et al., 2012; D’Souza, 2021; Kaufman et al., 2013; Mozaffari, 2013; Vaezi & Rezaei, 2019; Zedelius et al., 2019). However, rating hundreds or thousands of narratives is inherently subjective and incurs significant labor costs, which poses a major challenge to scientific progress and real-world application. Given the limitations of human scoring, researchers are increasingly exploring whether creativity assessment can be automated (Beaty et al., 2022; Beaty & Johnson, 2021; Dumas et al., 2020; Heinen & Johnson, 2018; Kenett, 2019)—yet such tools do not yet exist to assess the creativity of narrative texts.

Developing a reliable and automated metric that captures creativity in narrative text has potentially far-reaching and consequential implications. Creativity is among the most valuable attributes in the US workforce, and consequently, automated assessment of creativity is a top priority (Florida, 2014; Lichtenberg et al., 2008). Identifying the key components of creativity in narratives interests a broad array of researchers and practitioners including psychologists (D’Souza, 2021; Zedelius et al., 2019) and linguists (Mozaffari, 2013), as well as employers (Florida, 2014), educators (Graham et al., 2002; Vaezi & Rezaei, 2019), creative writers (Bland, 2011), and other practitioners (Barbot et al., 2012). The goals of the current paper are to (1) develop a new conceptualization of one component of creativity in narratives by integrating creativity theory and distributional semantics theory, (2) examine the psychometric properties of this new construct across diverse narrative texts and diverse participants, and (3) maximize accessibility by providing a tutorial and access to automated assessment of this construct with an open-source web application. The new construct is termed *divergent semantic integration* (*DSI*). It is the extent to which a narrative connects divergent ideas.

## Distributional semantics theory

The core principle of distributional semantics theory is that “you shall know a word by the company it keeps” (Firth, 1957, p. 11). Thus, words that tend to occur in the same contexts have similar meaning. For example, the words *teacher* and *educator* often co-occur with the words *student*, *classroom*, and *school*, and consequently have similar meaning according to distributional semantics theory. A word’s distribution or co-occurrence with other words across a large corpus (i.e., body of text) determines its meaning. By exploiting the statistical regularities in word co-occurrence patterns in large corpora, each word can be represented by a high-dimensional numerical vector. These numerical word representations are referred to as word vectors or word embeddings (Günther et al., 2019; Lake & Murphy, 2021). While these values do not have symbolic meaning themselves, they can be used to derive semantic similarity between words and texts. Leveraging semantic similarity from these models has opened an exciting new frontier of research in psychological science and beyond, including in attitudes and emotions (Caluori et al., 2020; Eichstaedt et al., 2021; Vo & Collier, 2013), cultural similarities and differences (Jackson et al., 2021), creativity (Beaty & Johnson, 2021; Dumas et al., 2020; Gray et al., 2019; Green, 2016; Heinen & Johnson, 2018; Johnson et al., 2021; Prabhakaran et al., 2014), and more (see Jackson et al., 2021, and Lake & Murphy, 2021, for recent reviews).

The way in which a distributional semantic model captures the statistical regularities in word co-occurrence patterns has important implications for how well its output (e.g., word embeddings) aligns with human judgments and for its plausibility as a model of human cognition (Kumar, 2021; Lake & Murphy, 2021). One of the first distributional semantic models employed latent semantic analysis (LSA; Landauer & Dumais, 1997). LSA is a *count* model (Baroni et al., 2014; although see Kumar, 2021) because it begins with a large word–document matrix and a frequency count of each word in each document. Context is defined as a document, where a document could be a news article or textbook chapter. The matrix then undergoes a number of mathematical transformations including singular value decomposition to generate a smaller number of latent dimensions (e.g., 300). This dimensionality reduction step is what allows global or indirect relations between words to be represented, even if the words did not co-occur directly in the documents. While remaining one of the most commonly utilized models, LSA’s plausibility as a model of human cognition is low, as it does not allow for incremental learning and requires an enormous word–document matrix of word counts as a starting point, far exceeding human memory capabilities (Hoffman et al., 2018). LSA often shows the lowest correlation with human judgments of semantic relatedness in comparison with other models (Mandera et al., 2017).

Another class of models, referred to as *predict* models (Baroni et al., 2014), attempt to predict a missing target word given its surrounding context, which could be 2–10 words or more. Among the most popular predict models is the continuous bag-of-words model (CBOW; Mikolov et al., 2013), which is considered a feed-forward neural network model because a sliding window (e.g., five words to the left and five words to the right of a masked word) moves through a corpus from beginning to end. CBOW consists of an input layer, output layer, and hidden layers. The weights of the model are updated iteratively to minimize the error between the target’s output and network’s output. CBOW has been quite successful at predicting human judgments of semantic relatedness (Mandera et al., 2017) and is considered more plausible as a model of human cognition than LSA, given its iterative error-reduction learning mechanism (Kumar, 2021; Lake & Murphy, 2021; Mandera et al., 2017).

A high-performing hybrid model with count and predict model properties, called Global Vectors (GloVe; Pennington et al., 2014), starts with a word-by-word co-occurrence matrix but tries to predict the co-occurrence values using a regression model similar to the stochastic gradient descent used in CBOW. It performs comparably to or better than CBOW in its prediction of human relatedness judgments (Baroni et al., 2014) and seems particularly sensitive to higher-order or indirect semantic relationships between words (Pennington et al., 2014).

One of the main limitations of LSA, CBOW, and GloVe is that the word embeddings they output are *context-independent*. This means these models have identical representations for homonyms (e.g., “bank”) regardless of the context in which they are used. In addition, context-independent word embeddings do a poor job representing polysemy, that is, the difference in word meaning across different contexts (Klein & Murphy, 2001). Taking a leap forward, *language models*, such as Google’s Bidirectional Encoder Representations from Transformers (BERT; Devlin et al., 2019) or the *Generative Pre-Trained Transformer* 3 (GPT-3; Open AI), use multiple layers of attention and position information to generate *context-dependent* word embeddings. For example, BERT generates two different word embeddings for the word *bank,* depending on whether it is in a sentence discussing a “river *bank*” or “robbing a *bank.*”

BERT’s superior sensitivity to context is due, in part, to its “self-attention” mechanism, which allows each word’s representation to influence the others in a sentence, to decide how much weight should be given to each word. That way, in a sentence about a “river bank,” the word *river* can heavily weight the representation of the word *bank* to disambiguate *bank’s* representation. The self-attention mechanism, along with BERT’s sheer complexity with 340 million parameters (i.e., BERT-large), enables highly nuanced estimates of word representations that reflect the richness of contextual meaning arising from syntax, word order, and word choice (Clark et al., 2019), distinguishing it from LSA, CBOW, and GloVe models that either de-emphasize or ignore these text elements. Consequently, BERT and other transformer-based models outperform models that generate context-independent word embeddings in their agreement with humans in question answering, sentence completion, and entity recognition (Devlin et al., 2019). Like the other models, BERT does not represent a process model of human cognition (Kumar, 2021; Lake & Murphy, 2021), but it is among the best distributional semantic models to date at capturing complex language understanding including nuanced semantic meaning (i.e., polysemy; Jawahar et al., 2019) and syntactic information such as determiners, objects of verbs, and co-referents (Clark et al., 2019; Jawahar et al., 2019).

## Creativity theory

Psychologists have been studying creativity for over half a century (e.g., Guilford, 1956; Kaufman & Beghetto, 2009; Silvia et al., 2009)^1^. Several theories have been proposed to explain the cognitive systems that support creative thinking. Among the most durable theories in the literature is the associative theory proposed by Mednick (1962), published 60 years ago in *Psychological Review*. According to associative theory, creativity involves making connections between remote concepts stored in memory, and individual differences in creativity can be attributed to variation in the strength of associations between concepts. Thus, a less creative person has strong associations between common connections (e.g., table–chair), and weak associations between uncommon connections (e.g., table–splendid). With a more creative person, in contrast, common and uncommon connections are of similar strength, which presumably makes it easier for them to overcome dominant associations (e.g., table–chair) in favor of more efficiently connecting remote associations (e.g., table–splendid). Mednick referred to these associative profiles in terms of hierarchies, with steep hierarchies characterizing less creative individuals and flat hierarchies marking more creative people. Despite its popularity, direct evidence for associative theory was lacking for many years, due in part to methodological challenges in modeling human memory.

More recently, computational methods have been applied to test classic assumptions of associative theory (e.g., Kenett, 2019; Olteţeanu et al., 2018). Network science is increasingly used to model the organization of concepts in semantic memory (Hills & Kenett, 2021), providing a means to quantify the strength of semantic associations in individual participants. Kenett and colleagues have shown that, compared with less creative individuals, highly creative individuals—people who perform well on psychometric tests of creativity—show semantic networks that are more densely connected, with high connectivity between concepts, shorter path distances, and less rigidity/structure (Christensen et al., 2018; Kenett & Faust, 2019; Li et al., 2021). This network organization is consistent with some predictions of associative theory regarding individual differences in creativity. Notably, however, the notion of semantic memory as a network was only later proposed by Collins and Loftus (1975), and Mednick’s (1962) theory predates this now widely accepted view—highlighting the need to update this classical creativity theory with contemporary advances, particularly in light of theoretical developments in semantic memory as a dynamic system (Kumar, 2021; Yee & Thompson-Schill, 2016).

Indeed, several theories of the creative process have been advanced over the years, including the Geneplore (generate-explore) model (Finke et al., 1992), Blind Variation and Selective Retention (BVSR; Campbell, 1960), and the dual pathway to creativity model (Nijstad et al., 2010), among others (see Abraham, 2018, for a comprehensive overview). Key to most theories is that creative thinking involves at least two steps: a generative step and an evaluative step. During idea generation, candidate ideas are explored via spontaneous associative processes; during idea evaluation, such candidates are scrutinized for their utility/appropriateness and elaborated upon accordingly to meet specific creative goals (Beaty et al., 2016). These creativity theories broadly map onto dual-process models of human cognition via type 1 (spontaneous/automatic) and type 2 (deliberative/controlled) processes (Sowden et al., 2015). Empirical support for dual process theories of creativity has come primarily from studies of individual differences, demonstrating contributions of associative and controlled cognitive abilities to performance on creative thinking tasks (Benedek & Jauk, 2018; Volle, 2018).

Individual differences research has benefited from advances in theories that can accommodate both associative and controlled cognitive abilities. Regarding controlled abilities, Nusbaum and Silvia (2011) provided an earlier demonstration that individual creative ability, assessed via performance on divergent thinking tasks, is related to individual differences in executive control (e.g., goal-directed switching between conceptual categories). Several studies have since provided additional evidence for the contribution of individual differences in cognitive control to creative performance, extending classical findings on the role of intelligence in creativity with mechanistic insight into why intelligence predicts performance on creative thinking tasks (Benedek et al., 2014; Frith et al., 2021; Gerwig et al., 2021; Weiss et al., 2021; Zabelina et al., 2019).

These findings have informed recent theoretical developments on individual differences in creative ability, such as the minimal theory of creative ability (MTCA), which proposes that two cognitive abilities are essential for optimal creative performance: intelligence (i.e., domain-general cognitive ability) and expertise (i.e., domain-specific knowledge; Stevenson et al., 2021). MTCA can explain a range of findings in the creativity literature through its minimalist framework, accommodating both general cognitive abilities (e.g., memory, reasoning) and sources of expert knowledge (e.g., writing short stories, teaching plot development) in explaining individual differences in creative performance (e.g., creative writing). Other theories have recently been proposed to conceptualize individual differences in creativity (e.g., Corazza & Lubart, 2021), with a focus on understanding people’s ability to connect remote concepts to form new and valuable ideas and products across domains.

## Integrating creativity theory and distributional semantics theory

We see an opportunity to expand creativity theories by leveraging recent advances in distributional semantics. Distributional semantics theory provides both a theoretical and computational framework for testing classical theories of creativity (e.g., associative theory) that have proved challenging to rigorously evaluate, as well as to test new theories on the role of semantics in creative thinking. Indeed, an increasing number of studies have begun to deploy distributional semantic models to study associative cognition and its contribution to explaining individual creativity. Gray et al. (2019) and others (Beaty et al., 2021) have used distributional semantic models to model performance on chain free-association tasks, using an approach called “forward flow” to quantify how far people travel in semantic space when generating free associations. Psychometric evaluation of distributional semantic models, applied to the forward flow task, has shown correlations with other measures of creativity (e.g., creative achievement and divergent thinking), pointing to their construct validity. Notably, Beaty et al. (2021) found that forward flow (assessed via distributional semantic models) predicts divergent thinking ability above and beyond general intelligence, supporting the view that associative abilities are a unique predictor of creative performance that is nonredundant with general cognitive ability. When applied to simple word association tasks, distributional semantic models have thus far allowed researchers to test the role of free association ability in creative thinking.

Distributional semantic models have also been used to automate the scoring of verbal creativity tasks, such as the classical alternate uses task (AUT) of divergent thinking (i.e., generating creative uses for objects). The AUT and other tests of creative thinking have historically required time-consuming and subjective scoring methods that can negatively impact their psychometric properties (e.g., via rater fatigue and disagreements on what constitutes a creative idea). In a recent paper, Beaty and Johnson (2021) found that distributional semantic models yielded strongly positive correlations with human creativity ratings on the AUT (cf. Dumas et al., 2020) and other word association tasks, alongside convergent validity with other creativity measures (e.g., creative achievement). This work has demonstrated that distributional semantic models can be a powerful tool for automating creativity assessment, thus significantly contributing to psychometric creativity research by accelerating and standardizing the arduous process of human creativity evaluation.

In the present research, we aim to extend these promising findings by applying distributional semantic models to more complex, ecologically valid measures of verbal creativity: narrative text. Narratives are pervasive in everyday life, and they are often applied in high-stakes contexts, from job applications to college admissions essays. Yet there is currently no standardized way of detecting creativity in such texts. Distributional semantic models offer a means to address this critical issue while providing a computational window into the cognitive processes involved in narrative creativity. To write a creative short story, for example, writers must retrieve remotely associated concepts from semantic memory, connect them to create a cohesive and compelling storyline, and elaborate the story, among other processes. Distributional semantic models are well positioned to capture a writer’s ability to retrieve remotely associated concepts and integrate them in a story.

As a concrete example, consider that someone is prompted to write a creative story about sending a letter. If they write about sending a letter to their mother about the grandkids from the post office, then they’ve covered the idea of letter, grandmother, family, and post office. Distributional semantic models will generate a word embedding for each of those words. None of these ideas is particularly original and would frequently occur together in the same context, so each word embedding would be relatively similar and exhibit high semantic similarity. Critically, generating these words in this particular story indicates that the writer did not effectively integrate divergent ideas in semantic space. In contrast, if someone writes about a grandmother sending a digital letter over sub-light-speed channels to an alien species to prevent re-instigating a war over species subjugation, then the ideas of grandmother, letter, digital, alien, speed of light, space, war, and subjugation are covered. These ideas are more original and varied, and will rarely occur in the same context. Consequently, each word embedding from this story will be quite different and exhibit low semantic similarity between each other. Generating these words indicates that the writer was much more creative, integrating divergent ideas in semantic space. Put simply, these two stories differ in the *extent to which they connect divergent ideas.*

As the former example highlights, integrating creativity theory and distributional semantics allowed for a novel conceptualization of how creativity may be captured in narratives. We term this construct *divergent semantic integration (DSI)* because it represents the degree to which a text integrates divergent ideas from divergent contexts. A major advantage of deriving the *DSI* construct from distributional semantics is that it allows for a precise quantitative operationalization. We propose that averaging the semantic distances (converse of semantic similarity) between all words in a story parsimoniously captures the distance between ideas and, consequently, *DSI*. It also provides complete coverage of the story’s content. In accordance with the aforementioned story example about a grandmother writing a digital letter to an alien species, each word in the story is rarely used in the same context, so computing the semantic distance between each word will result in high semantic distance values, and a high *DSI* score. See Eq. 1, where *n* is the number of words in a story, ω and κ are two word embeddings in the story, and *Dcos* is the cosine semantic distance between those two word embeddings.

1 $${\displaystyle \begin{array}{c}\sum\limits_{i=1}^{\left(\begin{array}{c}n\\ {}2\end{array}\right)}\frac{Dcos\left(\omega, k\right)}{n}\\ {} Dcos\left(\omega, k\right)=1-\frac{\omega \bullet \kappa }{\left\Vert \omega \right\Vert \left\Vert \kappa \right\Vert}\end{array}}$$

*DSI* is distinguishable from but related to various definitions and operationalizations of semantic diversity or semantic distinctiveness (Cevoli et al., 2021; Hoffman et al., 2013; Johns, 2021). For example, Hoffman et al. (2013) proposed a new measure of semantic diversity with the primary goal of capturing a new lexical characteristic of words. Hoffman et al. (2013) proposed measuring the degree to which a single word has diverse interpretations depending on the contexts in which it is used (i.e., polysemy). Note that this differs from the goal in the current paper, which is to capture the degree to which an entire narrative integrates semantically divergent ideas. Hoffman et al. (2013) quantified semantic diversity of a target word by using LSA to generate context embeddings that contained 1000 words and then computed the average of the cosine semantic distance between all contexts in which a word appeared. While it is an innovative approach, current evidence suggests that LSA may not generate reliable compositional context embeddings (Kumar, 2021; Luke & Murphy, 2021), and Cevoli et al. (2021) demonstrated that Hoffman’s et al.’s (2013) measure of semantic diversity did not reliably capture the degree to which a word is polysemous. Developing a closely related construct, Johns (2021) generated novel measures of contextual diversity that used word-context similarity in a distributional semantic model to ensure that a word’s contextual diversity score reflects the diversity of contexts in which the word appears. Johns’ measures of contextual diversity exhibit an advantage in the prediction of lexical decision reaction time and accuracy. These instantiations of contextual diversity are distinguishable from the *DSI* construct, because Johns’ (2021) primary goal was to develop a lexical characteristic of a single word that reflects the diversity of contexts in which it typically occurs, whereas *DSI* is designed to capture the degree to which a text connects divergent ideas in a full narrative.

There is some preliminary validity evidence for using the distance between ideas to capture creativity. Using a creative word association task, Johnson et al. (2021) showed that the average semantic distance between each creative idea a participant generated was correlated with human perceptions of both creativity and idea diversity, i.e., the diversity of contexts from which ideas are generated. However, it is not yet known whether this operationalization can be applied to narratives, which distributional semantic model is best, or whether it has desirable psychometric properties.

## Human assessment of creativity in narratives

In a comprehensive review, D’Souza (2021) highlights the strengths and weaknesses of the current methodological approaches to assessing creativity in writing, such as the consensual assessment technique (CAT) and rubric-based approaches. The advantage of the CAT (Amabile, 1982), where domain-specific experts rate each creative story, is its popularity and validity (Kaufman et al., 2013). However, a significant limitation is that expert judges do not articulate the criteria they use to assess creativity, making it difficult to identify key components (D’Souza, 2021). Critically, the best open science and replicability practices are hampered unless all aspects of the rating process are transparent (Nosek et al., 2015). In addition, the labor cost associated with using expert judges can be excessive for researchers and educators, hindering scientific progress and educational application.

The CAT also highlights the critical question of who decides what is creative in writing (Kaufman & Baer, 2012). For example, sociocultural context plays a role in what is interpreted as creative by the judges, the content of the writer’s work, and the identities of the creative writers (Alhusaini & Maker, 2015; see Hennessey et al., 2008, for evidence of the cross-cultural applicability of the CAT). Although there are many discipline-specific answers to the question of who should decide what is creative, for some domains (e.g., short stories), experts may not be needed to obtain adequate inter-rater reliability (Kaufman et al., 2009; Kaufman et al., 2013; but see Kaufman et al., 2008). In addition, even if a field were to agree on a set of core components of creativity in writing and nonexperts could be used as judges, the significant issues of labor cost, time-intensiveness, and lack of standardization remain.

One strength of a rubric-based approach is that it requires identification of specific criteria used to assess creativity in writing (Lubart et al., 2011; Mozaffari, 2013; Vaezi & Rezaei, 2019; Zedelius et al., 2019). However, rubrics are often vague, are open to interpretation, and require substantial training (D’Souza, 2021). To conclude, even with successful implementation of rubric-based assessment, human creative writing assessment remains subject to issues of labor cost, time-intensiveness, and lack of standardization. Automated approaches to creativity assessment offer a promising solution to many of the above issues.

## Automated assessment of creativity in narratives

Automating the assessment of creativity in writing has many advantages. An algorithmically derived creativity score can be efficiently produced, is easily replicable, and requires no time or effort in gathering human ratings (see “General discussion” for limitations). Over the past couple of decades, automated assessments have shown impressive validity in capturing writing characteristics such as grammar and text cohesion (Boyd et al., 2020; Crossley et al., 2016; McNamara et al., 2014; Pennebaker & Stone, 2003; Tausczik & Pennebaker, 2010). Only recently have these metrics been used to capture creativity (Skalicky et al., 2017).

The development of automated assessments of creativity in writing is in its early stages. Zedelius et al. (2019) asked participants to write a short creative story which was then scored by humans using a rubric. The rubric identified three key components in creative writing, including imagery, voice, and originality, with originality defined as the degree to which the story idea or plotline was original and unlike other stories. The creative stories were also scored by a number of automated metrics, including Coh-Metrix (McNamara et al., 2014) and LIWC (Linguistic Inquiry and Word Count, Pennebaker et al., 2015) indices. Although some indices explained variance in imagery and voice, neither Coh-Metrix nor LIWC metrics explained meaningful variance in originality.

Toubia et al. (2021) developed a number of novel computational metrics to predict how highly humans rated movies, TV shows, and books. Their distributional semantic model-based metrics were derived from TV and film scripts in addition to full text from novels. These metrics predicted web-scraped overall ratings from the Internet Movie Database (IMDb) and Goodreads, although correlations were generally weak. For example, an increase of one standard deviation in one metric was associated with an increase of 0.048 points on a 10-point Likert scale. There are many potential reasons for this low predictive power, including noise in the human ratings on IMDb and Goodreads, and semantic model and corpus choices, among others. Critically, Toubia et al.’s (2021) goal was not to capture creativity in stories, but rather to capture success as defined by crowd-sourced human ratings. Toubia et al.’s (2021) innovative and promising work highlights the utility of applying distributional semantic modeling to creative stories. However, before these automated metrics can be considered key components of creative writing, higher predictive power is needed.

## Present research

Across 27 different creativity prompts, we examined whether *DSI* is a key component of creativity in writing using human creativity ratings and other creativity measures as criteria. The choice of semantic model and the corpus on which *DSI* is computed are critical to its validity (Beaty & Johnson, 2021; Mandera et al., 2017). We compared six state-of-the-art distributional semantic models that vary in their computational architecture and corpora, including a count model approach (e.g., LSA, Landauer & Dumais, 1997), a predict model approach (e.g., CBOW, Mikolov et al., 2013), a count-predict hybrid model approach (e.g., GloVe, Pennington et al., 2014), and a transformer-based approach (i.e., BERT, Devlin et al., 2019). We also investigated *DSI*’s generalizability across individuals identifying as White and L1 English-speakers and Hispanic and L2 English-speakers, comparing *DSI* against human creativity ratings. We provide an open-source web application for computing *DSI* (osf.io/ath2s) and a step-by-step tutorial (https://osf.io/ath2s/) that is accessible to researchers and practitioners (e.g., educators).

## Study 1

In the first study, participants were given a three-word prompt and asked to incorporate all three words into a very short creative story (Prabhakaran et al., 2014). The primary goals in Study 1 were to examine the validity of *DSI* in its relation to human ratings of creativity in short stories, convergent validity (e.g., verb generation task), criterion-related validity (e.g., openness to experience), and its incremental validity above and beyond common lexical characteristics (e.g., word count, word frequency). Moreover, given past work on the role of intelligence in creativity (Stevenson et al., 2021; Taylor & Barbot, 2021), we examined whether intelligence facets theoretically relevant for creative writing (fluid and crystallized intelligence, broad retrieval ability) similarly correlate with *DSI*.

### Method

All materials, code, and analysis scripts are available on the Open Science Framework (OSF) (https://osf.io/ath2s/). None of the studies in the current paper was preregistered.

### Participants

Participants were 179 undergraduate students from Penn State University (*M*_*age*_ = 18.15, range = 18-26, 133 men, 46 women). They received course credit for their participation.

### Materials

The study was part of a larger project on individual differences in creative thinking and cognitive ability. Participants completed measures of verbal creativity, personality, and intelligence.

#### Five-sentence creative story

Participants completed the five-sentence creative story task (Prabhakaran et al., 2014). They were given the three-word prompt, *stamp-letter-send*, and asked to include all three words when writing (typing) a short story about five sentences in length. Five trained undergraduates evaluated the creativity of each story using the subjective scoring method (Silvia et al., 2008), which is based on the consensual assessment technique (Amabile, 1982). They were asked to evaluate each story on a scale of 1 (*very uncreative*) to 5 (*very creative*). The stories were 59.75 words in length on average (*SD* = 20.17, range = 16–104).

#### Verb generation task

To assess participants’ ability to generate creative word associations, we administered an abbreviated version of the verb generation task (Prabhakaran et al., 2014). In this task, participants are presented with a noun and asked to “think creatively” when coming up with a single verb that could be associated with a given noun. Verb responses are scored for creative quality using distributional semantic modeling (see Supplemental Material for more detail).

#### Crystallized intelligence (Gc)

Participants completed a measure of vocabulary knowledge to assess Gc: the extended range vocabulary test (24 items, 4 minutes) from the Educational Testing Service (ETS) Kit of Factor-Referenced Cognitive Tests (Ekstrom et al., 1976). The task presents a target word and asks participants to select a synonym of the target from a list of possible answer choices (omega = .55).

#### Fluid intelligence (Gf)

Participants completed a measure of matrix reasoning to assess Gf: the series completion task from the Culture Fair Intelligence Test (Cattell & Cattell, 1961/2008). The task shows three small images that change based on a given rule, and participants select the fourth image that correctly follows the rule (omega = .35).

#### Broad retrieval ability (Gr)

Participants completed a measure of verbal fluency to assess Gr: retrieving exemplars from the animal category (2 minutes). They were asked to “write down [type] as many animals as you can.” Duplicates and inaccurate responses were removed automatically using the semantic network and fluency utility (SNAFU; Zemla et al., 2020).

#### Openness to experience

Participants completed the openness to experience subscale of the NEO-FFI-3 (12 items; McCrae & Costa Jr, 1997). Openness is defined by a preference for fantasy, aesthetics, and intellectual engagement, and it is among the most robust predictors of creativity (Oleynick et al., 2017). Participants responded to a series of statements (e.g., “I have a lot of intellectual curiosity”) using a 1 (*not at all*) to 5 (*very much*) scale. The scale had adequate reliability (omega = .78).

### Automated assessment

#### Semantic models

Six semantic models were selected for comparison to maximize validity and generalizability using the following criteria: (1) pre-existing validity evidence showing associations between semantic distance and human judgments of semantic meaning and/or creativity (Beaty & Johnson, 2021; Devlin et al., 2019; Mandera et al., 2017), (2) variation in computational architecture, and (3) variation in text corpora used in the computational model (e.g., textbooks, Wikipedia, film subtitles).

Three semantic models were built using CBOW, based on algorithms from word2vec (Mikolov et al., 2013). These are termed predict models (Mandera et al., 2017). One semantic model, *cbowsubtitle*, is built on a corpus of subtitles (~385 million words); a second model, the *cbowukwac* model, is built on a concatenation of the subtitle corpus and a web crawling corpus (~2 billion words; see Ferraresi et al., 2008, for more details on corpora). Both of these semantic models has a window size of 12 words, 300 dimensions, and the most frequent 150,000 words (for source of the spaces and more details, see Mandera et al., 2017). The third CBOW semantic model, *cbowwiki*, was built on a concatenation of the British National Corpus (~2 billion words), the web crawling corpus, and the 2009 Wikipedia dump (~800 million tokens) with a window size of 11 words, 400 dimensions, and the most frequent 300,000 words (see Baroni et al., 2014, for source and more details).

The fourth and fifth semantic models are considered count models. The fourth model is one of the earliest models and uses LSA on the Touchstone Applied Science Associates (TASA) corpus (~100,000 words and 300 dimensions, Forster & Dunbar, 2009; Landauer et al., 1998; see also Günther et al., 2015, and the *lsa.colorado* interactive website for sources). The fifth model is called the GloVe (Global Vectors for Word Representation) model because it is particularly sensitive to global information across the entire corpus (Pennington et al., 2014). It was built on a concatenation of a 2014 Wikipedia dump and the gigaword corpus (~ 6 billion tokens), with 300 dimensions and the most frequent 400,000 words.

The sixth model is the Bidirectional Encoder Representations from Transformers (BERT) model, which advanced the field of natural language processing in a number of important ways (Devlin et al., 2019). In contrast to the previous five models, BERT generates context-dependent word embeddings, as discussed in the introduction. Although word embeddings are extracted from BERT, it is fit at the sentence level in order to account for context. It was built on a concatenation of the BooksCorpus (800 million words) and a Wikipedia extraction (2.5 million words, see Devlin et al., 2019, for more).

#### Story preprocessing

For all semantic models except BERT, commonly used words (stop word list from *tm* package in R, Feinerer, 2012), numbers, and punctuation were removed because they can bias semantic distance scores (Forthmann et al., 2018). Regarding the BERT model, because all words and punctuation in a sentence can provide context, nothing (except some special characters) was removed before extracting word embeddings. Words were not stemmed (i.e., converted to word roots), as evidence is mixed on whether this preprocessing step improves agreement between semantic models and human judgments (Mandera et al., 2017). Finally, words were not spell-checked, as this can often require human judgment, and our goal was to automate creativity assessment. Misspelled words were treated as missing data. In one previous study, totaling the number of misspelled words and words that the semantic model did not recognize resulted in a 4.1% loss of data, which seems worth the labor savings (Johnson et al., 2021).

#### DSI

**Fig. 1:**
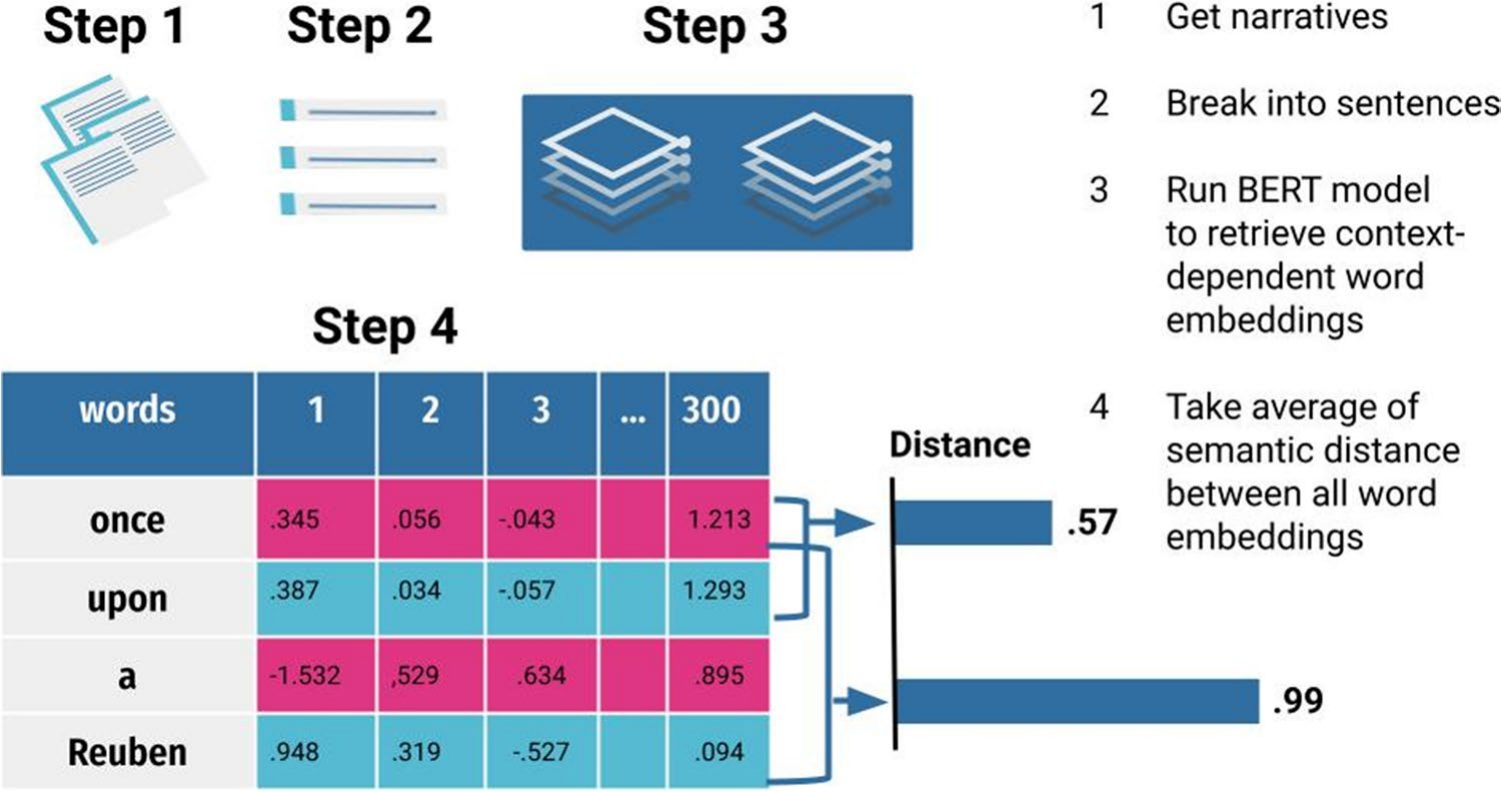
Computing DSI scores from the BERT model. The figure depicts the steps to computing *DSI* using the BERT model, but *DSI* may also be computed with semantic models that generate context-independent word embeddings (e.g., GloVe) with a slightly modified sequence of steps outlined below

See Eq. 1 for how *DSI* is computed. We provide a detailed tutorial with R code and Python code (osf.io/ath2s) to extract *DSI* scores. In addition, this code is incorporated into the *SemDis* web app (osf.io/ath2s; Beaty & Johnson, 2021) so that a user can upload a data file of stories and retrieve *DSI* scores automatically. The computational steps will be summarized briefly here (Fig. 1).

To extract context-independent word embeddings from the three predict models and two count models, the stories are first stripped of punctuation and stop words. Then, they are tokenized, which essentially means to separate into individual words or word pieces (e.g., “bath” and “ing” for bathing). Each word from the story is matched to its corresponding word vector in each semantic model. Next, the cosine semantic distance is computed between all word embeddings in each individual’s story, added up, and divided by the total number of word pairs in their story, resulting in a single *DSI* score for each story for each of the semantic models that generate context-independent word embeddings.

To extract context-dependent word embeddings from BERT, the stories are split into sentences, and word embeddings are uniquely generated depending on the context of the sentence. BERT generates 24 different word embeddings (i.e., 24 layers) for each word in each sentence in a story, each reflecting a unique set of weights that index how much priority each word should receive in the representation relative to every other word in a sentence. Thus, a single BERT model produces 24 different options of word embeddings from which to choose. Note that the previous models that generate context-independent word embeddings produce only one set of word embeddings, so there are no layers from which to choose. Determining how to utilize the rich information contained in these layers is a nontrivial decision without much prior literature for guidance. Using all layers is computationally expensive and not recommended (Devlin et al., 2019). We selected layers and determined how to combine them based on empirical guidance (i.e., determine which layers correlate most highly with human creativity ratings), some prior literature, and theoretical justification. Because *DSI* should quantify how well writers connect divergent ideas, we wanted the metric to maximize its coverage of semantic space and its ability to capture nuance in semantics based on how words are used in context. There is preliminary evidence that the early and middle layers in BERT are sensitive to syntactic and semantic information (Jawahar et al., 2019). In addition, the early to middle layers correlated most highly with human creativity ratings (see Supplemental Material for analysis of all 24 BERT layers). Consequently, we selected two early to middle layers (i.e., layers 6 and 7) and computed *DSI* by taking the pairwise cosine semantic distance between all word embeddings from both layers, instead of summing, averaging, or concatenating the layers.

### Procedure

Participants completed the study in groups of 2–6 on desktop computers (in private cubicles) running PsychoPy experiment software. After they had signed consent forms, participants were asked to complete the creative story task, self-report scales, and intelligence tasks.

### Results

#### Comparison of semantic models and relation to human ratings

**Fig. 2:**
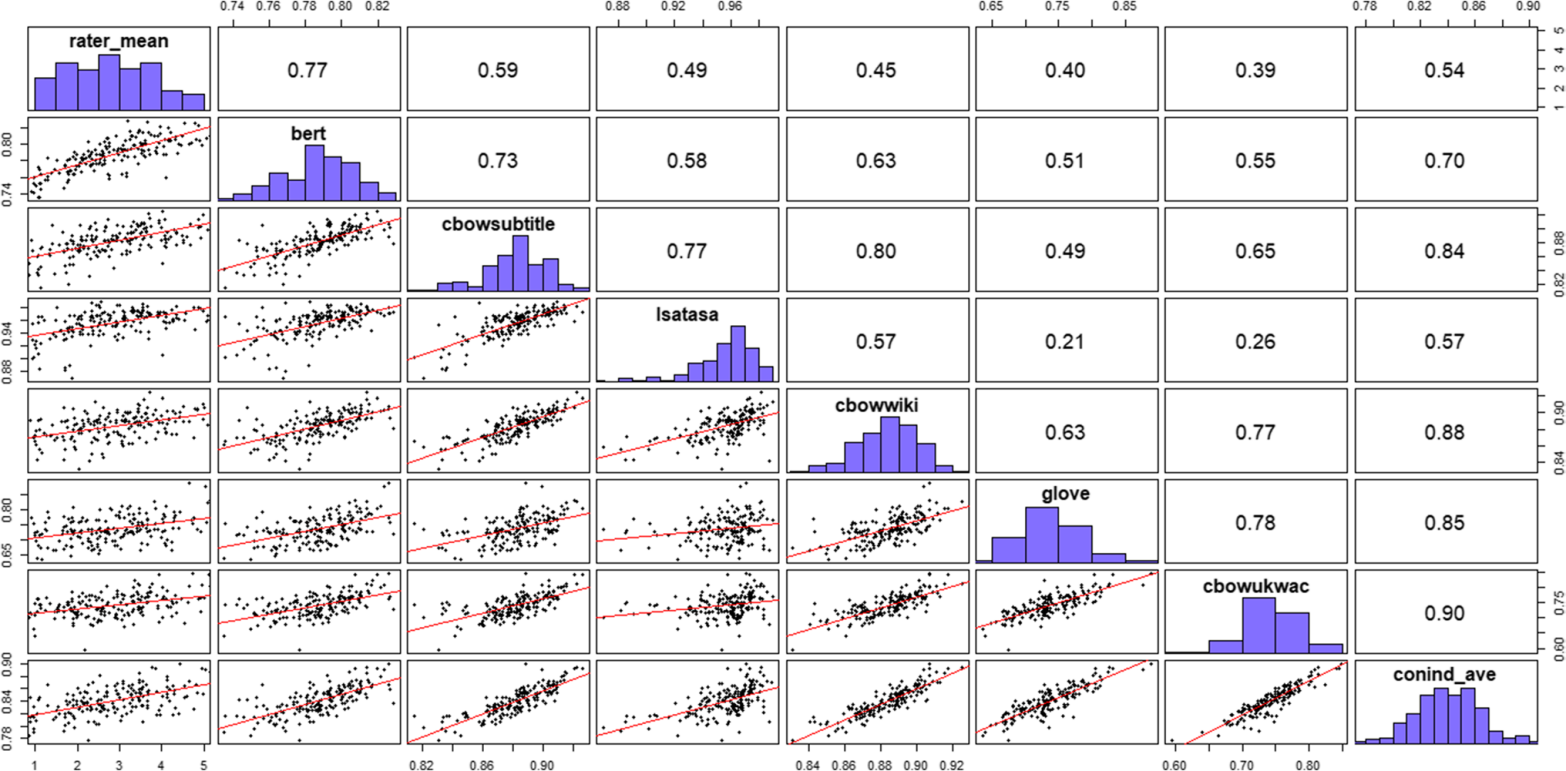
Study 1 Scatterplot matrix of all DSI models and human ratings. *N* = 179. The rater_mean is the mean of the creativity ratings from five human raters. All other labels refer to the semantic model from which each *DSI* score was generated. The conind_ave represents the mean score from the first five semantic spaces, which was previously the best-performing approach with other creativity tasks (Beaty & Johnson, 2021). The scatterplot matrix shows the relationship between all variables, with the scatterplots depicted in the space below the diagonal, the Pearson correlation coefficients in the space above the diagonal, and the univariate frequency distributions on the diagonal (generated with the *psych* R package, Revelle, 2022). Column names are on the diagonal, and the relationship between variable are the intersection of two columns

All analysis scripts and data files from all studies are provided on OSF (osf.io/ath2s). To determine the relationship between *DSI* and human ratings of creativity, we examined the correlation between each of the six *DSI* scores and the mean creativity score from five human raters. Figure 2 depicts a scatterplot matrix showing the relationship between all variables and depicts univariate frequency distributions.

**Fig. 3:**
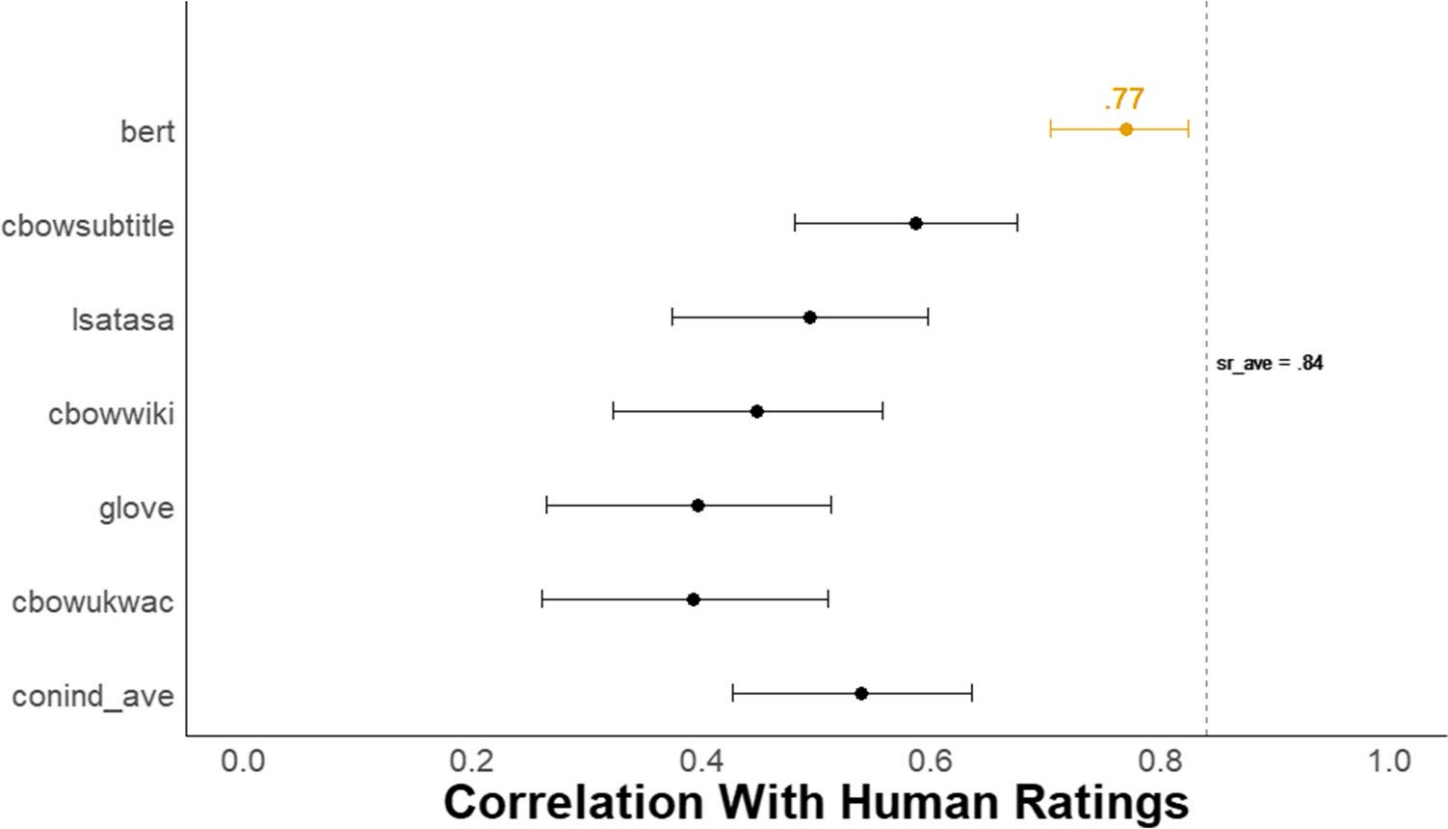
Study 1 Forest plot of correlation between all DSI models and human creativity ratings. *N* = 179. The dots represent the correlation between each *DSI* score and the average human creativity rating with 95% Cis. The dotted line, *sr_ave*, represents the average correlation between each single human rater and the average human creativity rating

Figure 3 shows a forest plot of the Pearson correlations with 95% confidence intervals between each of the six *DSI* scores and the mean creativity scores from human raters. The BERT model demonstrated a substantial advantage over all other models, with a correlation with human raters of *r* = .77, 95% CI [.70, .82]. BERT outperformed a model that averages the scores from five semantic models (i.e., conind_ave) that generate context-independent word embeddings (*r* = .54, 95% CI [.43, .64]; despite these models showing strong prediction of human ratings in prior work with other verbal tasks, e.g., Beaty & Johnson, 2021). The difference between these two correlations was reliable (difference in *r* = .23, 95% CI [.15, .32], *z* = 5.72, *p* < .001; Steiger, 1980, from the *cocor* package in R, Diedenhofen, 2016).

To compare BERT’s performance with that of a single human rater, we took the average correlation between each human rater’s score and the mean human creativity score across all raters (i.e., *sr_ave* for single-rater average, in Fig. 3). This way both the semantic models and each single human rater are compared with the same criterion—mean of human creativity ratings. The BERT model’s correlation with human raters was lower but not reliably different from *sr_ave* = .84, 95% CI [.80, .88] (difference in *r* = .07, 95% CI [−.003, .15], *z* = 1.88, *p* = .06; from the *cocor* package in R, Diedenhofen, 2016). This indicates that BERT *DSI* scores correlate with the mean of human creativity ratings about as well as the average human rater does. In the Supplemental Material, we also provide intra-class correlation coefficients (ICC) for both single-rater and average-rater measurement for assessments of overall human inter-rater reliability for all studies (see Table 7 in Supplemental Material).

#### Convergent and criterion-related validity

**Fig. 4:**
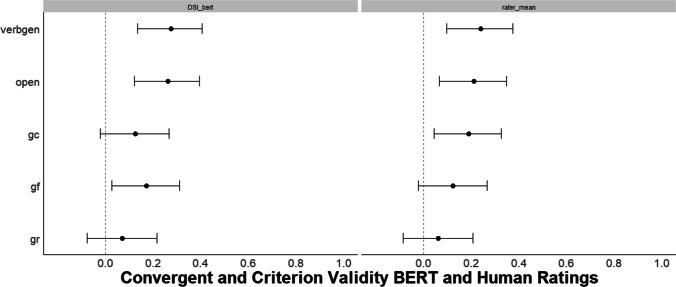
Study 1 Convergent and criterion-related validity of BERT DSI. *N* = 179. The left panel shows the correlations with 95% CIs between the best-performing *DSI* model (i.e., BERT) with the same convergent and criterion-related validity measures. The right panel shows the correlations with 95% CIs between the mean of human creativity ratings of the creative stories and each measure. verbgen = verb generation task, open = openness to experience, gc = crystallized intelligence, gf = fluid intelligence, gr = broad retrieval ability (verbal fluency)

Based on related work on semantic distance, correlations between *DSI* and convergent and criterion-related measures were in the expected range of *r* = .20–.30 (Fig. 4; Beaty & Johnson, 2021; Beaty et al., 2021, 2022; Prabhakaran et al., 2014). We found that the best-performing *DSI* model (i.e., BERT) demonstrated convergent and criterion-related validity by correlating with previously validated indices of creativity including the verb generation task, personality (openness to experience), and intelligence facets at levels comparable to human ratings of the same stories (see Supplemental Material for convergent and criterion-related validity with conind_ave). This pattern of results suggests that *DSI*, applied to a single creative story prompt, provides a valid index of creative ability.

#### Incremental validity

To determine whether BERT *DSI* explains unique variance in human creativity ratings above and beyond common lexical characteristics and indicators of vocabulary level (see Mandera et al., 2017, for a similar approach), we conducted a hierarchical regression, where lexical characteristics were entered first, followed by *DSI*, including total word count (Taylor et al., 2021), word frequency (Brysbaert et al., 2019), word prevalence (Brysbaert et al., 2019), age of acquisition (Brysbaert et al., 2019), readability (quanteda R package, Benoit et al., 2022; Kincaid et al., 1975), and a measure of text lexical diversity (MTLD; McCarthy & Jarvis, 2010). See Table 1, which shows that *DSI* explains substantial additional variance in human creativity ratings while controlling for lexical characteristics (Δ*R*^*2*^ = .132, 95% CI [.07, .20]; using *apaTables* R package, Stanley, 2021). Critically, only word count and *DSI* remain significant predictors with all predictors in the model. This indicates that *DSI* subsumes previous predictors and is not a simple reflection of vocabulary level or story length. The standardized effect of *DSI* (b^*^ = .63) was nearly double that of word count (b^*^ = .32), which has previously been shown to predict human creativity ratings in stories (Taylor et al., 2021).

**Table 1:** Study 1 Regression results using human creativity ratings as the criterion

Predictor	*b*	*b* 95% CI [LL, UL]	b*	b* 95% CI [LL, UL]	Fit
(Intercept)	−7.94	[−19.59, 3.71]			
word_count	0.03**	[0.02, 0.04]	0.57**	[0.45, 0.69]	
readability	0.01	[−0.04, 0.05]	0.02	[−0.10, 0.14]	
freq	−0.76	[−1.71, 0.19]	−0.11	[−0.25, 0.03]	
prev	5.28*	[1.25, 9.32]	0.14*	[0.03, 0.25]	
aoa	0.19	[−0.44, 0.82]	0.05	[−0.10, 0.19]	
mtld	0.01**	[0.00, 0.02]	0.19**	[0.06, 0.31]	
					*R*^*2*^ = .531**
					95% CI [.42,.60]
(Intercept)	−25.99**	[−36.80, −15.17]			
word_count	0.02**	[0.01, 0.02]	0.31**	[0.19, 0.43]	
readability	−0.02	[−0.06, 0.02]	−0.06	[−0.16, 0.04]	
freq	−0.43	[−1.24, 0.38]	−0.06	[−0.18, 0.06]	
prev	2.34	[−1.16, 5.84]	0.06	[−0.03, 0.15]	
aoa	−0.19	[−0.73, 0.35]	−0.04	[−0.17, 0.08]	
mtld	−0.00	[−0.01, 0.00]	−0.06	[−0.18, 0.06]	
DSI_bert	33.26**	[25.25, 41.28]	0.63**	[0.48, 0.79]	
					*R*^*2*^ = .663**
					Δ*R*^*2*^ = .132**
					95% CI [.07, .20]

*N* = 179. *b* represents unstandardized regression weights. b* indicates the standardized regression weights. *LL* and *UL* indicate the lower and upper limits of a confidence interval, respectively. * indicates *p* < .05. ** indicates *p* < .01

## Study 2

Study 1 provided strong evidence that a novice writer’s ability to connect divergent ideas, as assessed by *DSI*, is a key component of creativity in writing. The BERT model of *DSI* substantially outperformed five other semantic models, explaining nearly 60% of the variance in human creativity ratings, even approaching human inter-rater agreement. This is remarkable given that *DSI* represents only a single component of creativity. We also found convergent and criterion-related validity evidence—*DSI* correlated with established markers of creativity (openness and novel word association) to a similar magnitude as human ratings—as well as correlations with crystalized and fluid intelligence, consistent with past work implicating general cognitive abilities in explaining individual differences in verbal creativity (Frith et al., 2021; Gerwig et al., 2021; Nusbaum & Silvia, 2011; Stevenson et al., 2021).

The primary goal of Study 2 was to examine the strength of the relationship between *DSI* and human creativity ratings while minimizing prompt-specific and rater-specific variance, by deriving latent variables and using a confirmatory factor analysis (CFA) framework (Kline, 2015). In addition, given that participants were asked to write seven short stories, reliability was also estimated.

### Method

#### Participants

Using Amazon’s Mechanical Turk, 153 participants (*M*_*age*_ = 38.62; range = 22–70; 82 women, 68 men, 3 nonbinary; 97% English first-language; 78% White, 9% African-American, 3% Asian-American, 9% other) were recruited and compensated $5.00 for participation. This excludes 16 responses that were nonsense or single words (likely bots).

### Materials

*Five-sentence creative story task.* The stamp-letter-send prompt from Study 1 was used for replication purposes, and six additional three-word prompts were created. For three of those prompts, there was a high semantic distance between the words in the prompt (average semantic distance = .97, gloom-payment-exist, organ-empire-comply, statement-stealth-detect) and for four prompts, there was a low semantic distance between the words in the prompt (e.g., average semantic distance = .69, stamp-letter-send, belief-faith-sing, petrol-diesel-pump, year-week-embark), *t*(3.79) = 6.06, *p =* .004, *d* = 4.35, 95% CI [3.61, 29.9] (*cohens_d* function from the *rstatix* package, Kassambara, 2021). See Supplemental Material for more detail on prompt generation. The stories were 69.58 words in length on average (*SD* = 21.70, range = 12–168).

#### Procedure

Participants first completed demographics and then received all seven 3-word prompts, one at a time, in randomized order by participant. They were given four minutes to write each 4–6-sentence creative story that incorporated all three words. They were encouraged to use their imagination and creativity while writing the story. See Supplemental Material for instructions scripts and instruction check questions.

### Results

#### Creativity prompt comparison

**Fig. 5:**
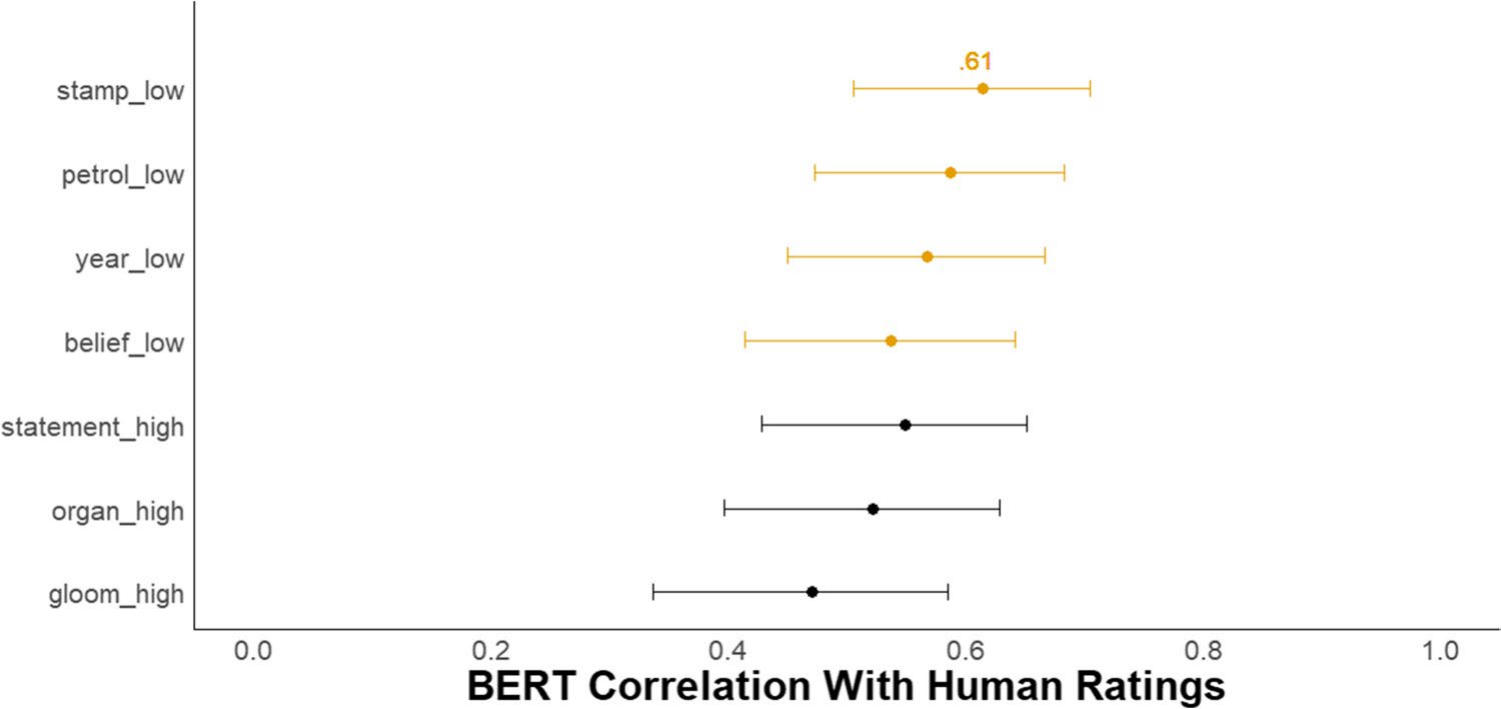
Study 2 Forest plot of correlation between BERT DSI models and human creativity ratings with seven prompts. *N* = 153. The dots represent the correlation between BERT *DSI* scores for each of seven story prompts and the average human creativity rating with 95% CIs. The low vs. high after the underscore refers to whether the semantic distance between the three words in the prompt was low or high, respectively

Given BERT’s superior performance in Study 1 for the five-sentence creative story task, and given that the same task is used in Study 2, we focus on BERT *DSI* scores. Figure 5 depicts a forest plot of the Pearson correlations with 95% confidence intervals between the BERT *DSI* scores and the mean creativity scores from human raters for the seven prompts. It shows that the prompts with low semantic distance between the words in the prompt had a slight advantage (compared with high semantic distance prompts), with the stamp-letter-send prompt exhibiting the highest correlation with human creativity ratings, *r* = .61, 95% CI [.50, .70]. The BERT model correlation with human raters for the stamp-letter-send prompt was reliably lower than the average correlation between each single human rater and the mean creativity rating (*sr_ave* = .89, 95% CI [.85, .92]; difference in *r* = .28, 95% CI [.18, .39], *z* = 6.17, *p* < .001). See Table 6 for *sr_ave* for all prompts and Supplemental Material for the ICCs for all prompts and forest plots for semantic model comparisons for each prompt and evidence of incremental validity.

#### Reliability

A single-factor model with the seven *DSI* scores from the BERT model (one indicator for each of the seven prompts) was used to compute an omega = .88, indicating excellent reliability (*semTools* R package, *reliability* function, Jorgensen et al., 2022).

#### Confirmatory factor analysis

**Fig. 6:**
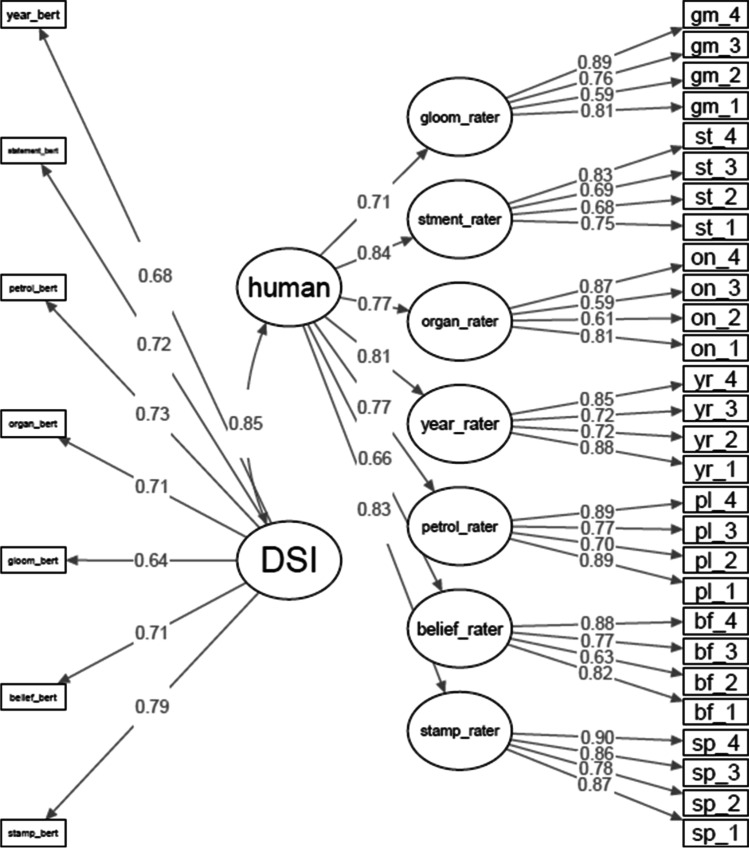
Study 2 Confirmatory factor analysis of DSI scores and human creativity ratings. *N* = 153, semdiv = *DSI* from BERT model latent variable, human = latent variable of human creativity scores, stamp_bert = BERT *DSI* score from the stamp-letter-send, stamp_rater = latent variable from four human raters of the stamp-letter-send prompt. Plot was created in *semPlot* R package (Epskamp et al., 2022)

CFA was conducted in which each human rater and each BERT *DSI* score for each prompt served as observed variables (Fig. 6). A single first-order latent variable was created from the seven *DSI* scores, and seven first-order latent variables were estimated from all four raters for each prompt. A second-order latent variable was created from the seven first-order latent variables representing human creativity ratings. All observed and latent variables were standardized before model fitting via robust maximum likelihood, and the variance of latent variables was set to 1. Note that due to the relatively small sample size, CFA results should be interpreted with caution.

Of primary interest was the latent variable correlation between the *DSI* metric and human ratings of creativity, which was very strong at *r* = .85, *p* < .001, 95% CI [.78, .91] (l*avaan* R package, Rosseel, 2022). Fit indices indicate a good model fit in the context of high standardized loadings (i.e., a strong measurement model; Heene et al., 2011; McNeish et al., 2018; McNeish & Wolf, 2021; Wolf et al., 2013), including root mean square error of approximation [RMSEA] = .058, 90% CI [.05, .066], standardized root mean square residual [SRMR] = .076, and CFI = .911. McNeish and Wolf (2021) developed a simulation-based tool to dynamically adjust fit index cutoff values (for adequate model fit) based on a model’s standardized loadings, degrees of freedom, and sample size. However, the tool does not yet provide estimates for higher-order models, like the one in the current study.

## Studies 3A and 3B

Study 2 demonstrated that when examining the common variance across creativity prompts, the correlation between *DSI* and human creativity ratings was very strong at *r* = .85, explaining over 72% of the variance. In addition, reliability (i.e., internal consistency) was excellent for *DSI*. The primary goal of Studies 3A and 3B was to determine whether *DSI* captures creativity in narrative text according to expert human ratings. In Study 3A, experts provided creativity ratings of another creative writing task—the storyboard creativity task—where images are used as prompts. In Study 3B, the correlation between *DSI* and human raters was compared across novice raters, quasi-expert raters, and expert raters with two different single-word creativity prompts. We expect there to be minimal differences across rater types, as previous work showed that the rank order agreement between all human rater groups was relatively high for ratings of short stories (Kaufman et al., 2013).

### Method

#### Participants

In a secondary analysis of Taylor et al.’s (2021) data, participants were recruited from a northeastern US university (*N* = 125; *M*_*age*_ = 21.36, *SD* = 4.41; 76 women, 44 men, 2 transgender, 3 prefer not to say; 58 White, 31 Hispanic-American, 25 multiple ethnic backgrounds, 19 Asian American, 19 Black/African American, 1 American Indian/Alaskan Native, and 3 prefer not to say).

### Materials

*Storyboard task.* Participants were given three black and white photographs and asked to write a story in which the three photographs were prompts for the beginning, middle, and end of the story (Taylor & Barbot, in press; Taylor et al., 2021). The stories from all prompts were 57.78 words in length on average (*SD* = 52.10, range = 2–434) and took a maximum of 21 minutes to complete.

#### Procedure

The participants completed stories for four different prompts, with no time limit (mean completion time for each prompt = 148.60 seconds). Three expert raters were used to evaluate the stories using the CAT, all of whom were professional creativity researchers. See Supplemental Material for more detail.

### Results

#### Comparison of semantic models and relation to human ratings

**Fig. 7:**
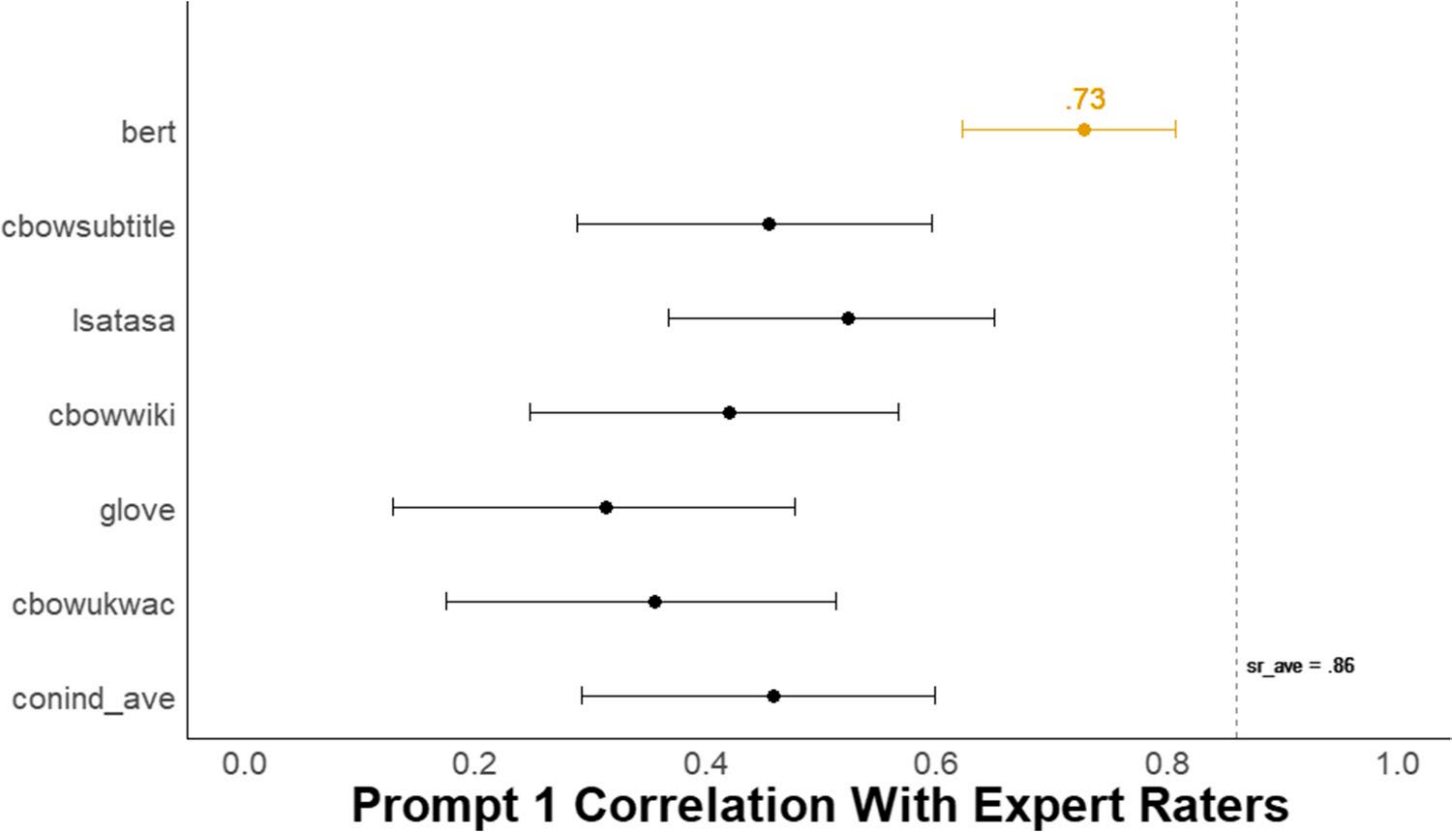
Study 3A Forest plot of correlation between all DSI models and expert human creativity ratings. *N* = 125. The dots represent the correlation between each *DSI* score and the average expert human creativity rating for the first storyboard prompt with 95% CIs. The dotted line, *sr_ave*, represents the average correlation between each single human rater and the average human creativity rating

As Fig. 7 shows, for the first storyboard prompt, the BERT model had a substantial advantage over all other models, with a correlation with human raters of *r* = .73, 95% CI [.62, .81]. BERT outperformed a model that averages scores from five semantic models (i.e., conind_ave) that generate context-independent word embeddings, *r* = .46, 95% CI [.29, .60]. The difference between these two correlations was reliable (difference in *r* = .27, 95% CI [.16, .40], *z* = 5.01, *p* < .001). See Supplemental Material for comparable results for the other three-image prompts. In addition, see Supplemental Material for incremental validity that, like Studies 1-2, shows substantial explanatory power for *DSI* above and beyond word count and other linguistic indices. Note that a CFA could not be reliably fit due to small sample size.

In addition, the BERT model correlation with human raters approached, but was reliably lower than, the average correlation for the first storyboard prompt between each single human rater and the mean creativity rating (*sr_ave* = .86, 95% CI [.80, .90]; difference in *r* = .13, 95% CI [.04, .23], *z* = 2.85, *p* = .004).

#### Reliability

A single-factor model with four *DSI* scores from the BERT model (one indicator for each of the four image prompts) was used to compute an omega = .75, indicating very good reliability.

## Study 3B

### Method

#### Participants

In a secondary analysis of data from Kaufman et al. (2013), the participants (*N* = 205) completed the study for course credit (*M*_*age*_ = 24.20, *SD* = 8.73; 151 women, 54 men; 75 White, 47 Asian-American, 37 Hispanic-American, 25 Black/African-American, 21 multiple ethnic backgrounds). Three participants’ stories were not rated by quasi-experts, so were excluded from analysis.

### Materials and procedure

The materials were created and used for a series of studies designed to look at the impact of different types of expertise on assigning creativity ratings (Kaufman et al., 2009; Kaufman et al., 2013) and a study of differences by ethnicity in rating creative work (Kaufman et al., 2010).

#### Short story task

Participants were asked to write a short story using one of two provided titles (“2305” and “Execution”). There were no other restrictions in terms of length or style. They were given a maximum of 10 minutes. The stories from all prompts were 194.23 words in length on average (*SD* = 172.26, range = 22–1893).

#### Raters

##### Expert raters

Ten expert raters were used to rate the stories for creativity. Five expert raters had MFAs in creative writing, three raters received PhDs in English, and the remaining two had significant experience in both publishing their own creative writing and assessing student writing.

##### Quasi-expert raters

There were four groups of quasi-experts. The first group consisted of 12 advanced undergraduate or master’s level students with some degree of experience in creativity research. The second group comprised nine English teachers actively working in the schools. The third group comprised 10 students who were at the time earning a master’s degree in English with the intent of becoming English teachers. The fourth group comprised 10 students who were at the time earning a master’s degree in education with the intent of becoming English teachers.

##### Novice raters

A group of 108 novices also rated all materials. See Supplemental Material for more detail.

### Results

#### Novice vs. quasi-expert vs. expert raters

**Fig. 8:**
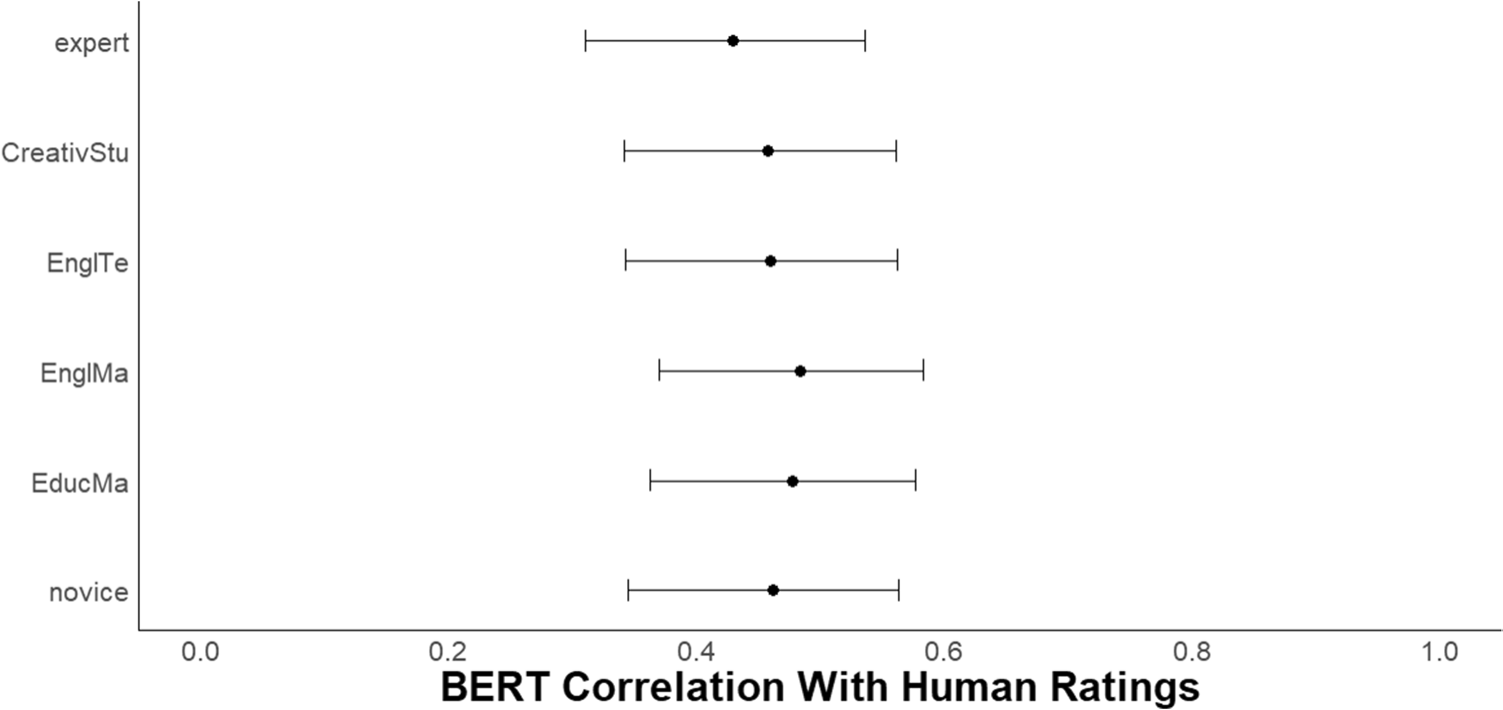
Study 5 Forest plot of correlation between DSI and human creativity ratings and all rater types. *N* = 202. The dots represent the correlation between the BERT *DSI* score and each type of human rater including experts, CreativStu = advanced student involved in creativity research, EnglTe = English teachers, EnglMa = English master’s students, EducMa = education master’s students, novice = undergraduate students from a large university

As Fig. 8 shows, there were no reliable differences in the correlations between BERT *DSI* and the mean of human creativity ratings across each rater type, with correlations ranging from .45 to .50. See Supplemental Material for a forest plot of semantic model comparisons and evidence of incremental validity.

### Studies 4A, 4B, 4C

Studies 3A and 3B showed that the strong relationship between *DSI* scores and human ratings extends to novice raters, quasi-expert raters, and expert raters. The goal of Studies 4A, 4B, and 4C was to determine whether *DSI* captures human creativity ratings across diverse creative story tasks, prompts, and story lengths. In Study 4A, we will examine the relation between *DSI* and human creativity ratings with the story ideas paradigm, where participants provided descriptions of setting, plotlines, and characters but did not write stories with full narrative—and did this for 10 different prompts. In Studies 4B and 4C, we investigated whether the strong relationship between *DSI* and human creativity ratings generalizes to longer stories.

## Study 4A

### Method

#### Participants

In a secondary analysis of data collected by Ward et al. (2013), participants were 86 undergraduate students who participated in the study for course credit (*M*_*age*_ = 19.43, range = 18–36; 48 women, 38 men; other demographic information unavailable).

### Materials and procedure

#### Story idea task

In response to adjective–noun prompts (e.g., hostile enemy), participants described what they would write about in the story instead of actually writing the story (Estes & Ward, 2002). They were told that the story ideas could be anything they wanted them to be: realistic, strange, funny, silly, practical, educational, or anything else that came to mind. They were also instructed to describe what the story was about, where it was set, who the characters were, and what would happen in the story. The story ideas from all prompts were 42.20 words in length on average (*SD* = 21.17, range = 4–139) and took a maximum of 45 minutes to complete all 10 story ideas. See Supplemental Material for all prompts and more detail. A total of eight quasi-experts rated the stories for creativity, including advanced undergraduate and master’s level students with some degree of experience in creativity research.

### Results

#### Comparison of semantic models and relation to human ratings

**Fig. 9:**
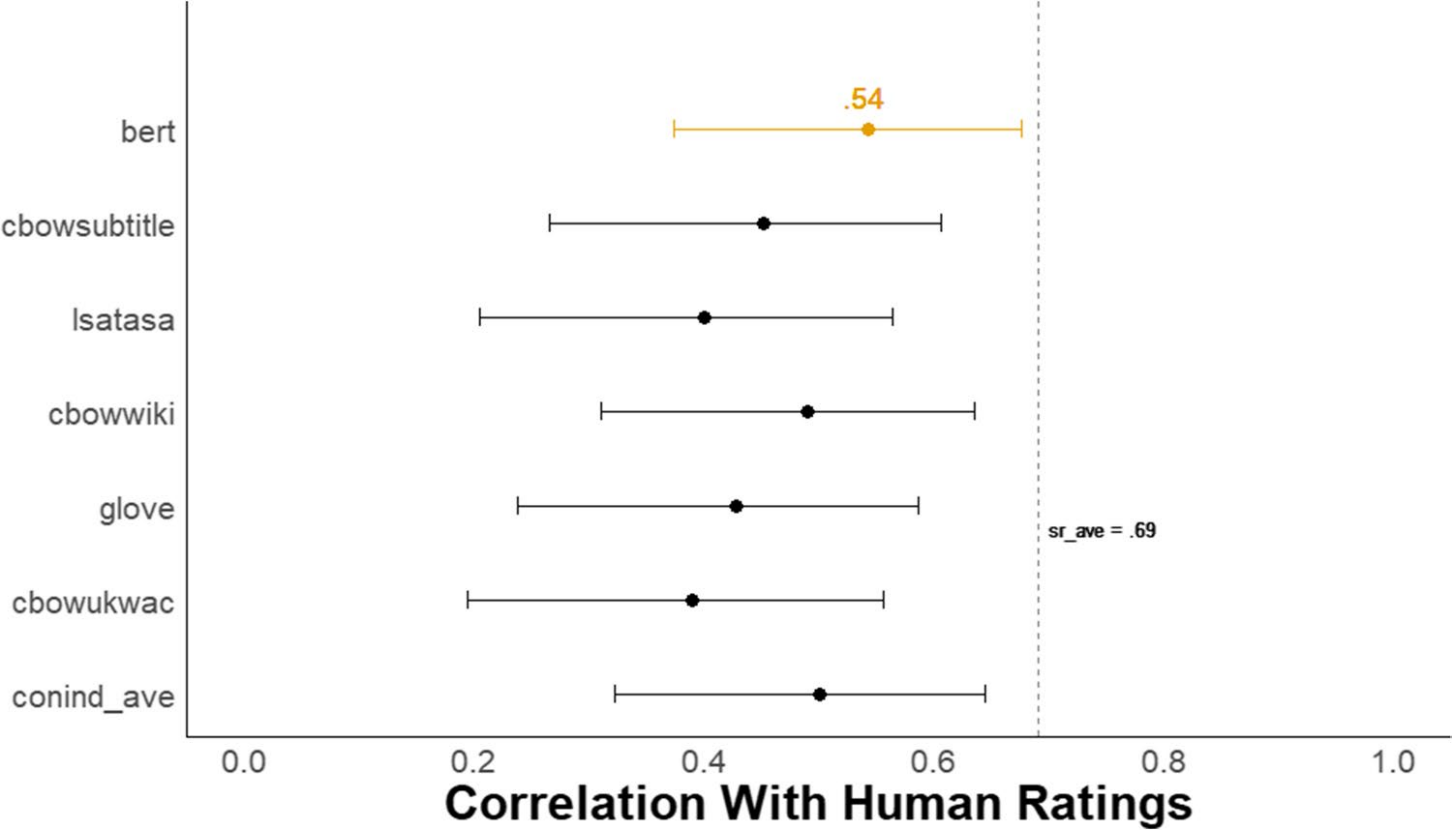
Study 4A Forest plot of correlation between all DSI models and human creativity ratings. *N* = 86. The dots represent the correlation between each *DSI* score and the average human creativity rating with 95% Cis for the death-living and death-tragic prompts (combined). The dotted line, *sr_ave*, represents the average correlation between each single human rater and the average human creativity rating

Figure 9 shows a forest plot of the Pearson correlations with 95% confidence intervals between each of the six *DSI* scores and the mean creativity scores from human raters for the *death-living and death-tragic* prompts (combined). The BERT model had an advantage over all other models, with a correlation with human raters of *r* = .54, 95% CI [.37, .68]. BERT outperformed a model that averages scores from five semantic models (i.e., conind_ave) that generate context-independent word embeddings, *r* = .50, 95% CI [.32, .64]. The difference between these two correlations was not reliable (difference in *r* = .04, 95% CI [−.12, .20], *z* = 0.490, *p* = .624). See Supplemental Material for comparable results for the other nine prompts and evidence for incremental validity. Note that a CFA could not be reliably fit due to small sample size.

In addition, the BERT model correlation with human raters approached the average correlation for the death prompt between each single human rater and the mean creativity rating (*sr_ave* = .69, 95% CI [.57, .79]; difference in *r* = .15, 95% CI [−.04, .35], *z* = 1.57, *p* = .116).

#### Reliability

A single-factor model with 10 *DSI* scores from the BERT model (one indicator for each of the 10 prompts) was used to compute an omega = .88, indicating excellent reliability.

## Study 4B

### Method

#### Participants

In a secondary analysis of data collected by Taylor and Kaufman (2020), participants (*N* = 163) participated for course credit (*M*_*age*_ = 23.88, *SD* = 6.60; 146 women, 17 men; 68 Hispanic-American, 41 White, 21 African-American, 18 multiple ethnic backgrounds, 10 Asian-American, 3 other, 1 Middle Eastern/Arab, 1 Native American).

### Materials and procedure

The stories were collected as part of a larger study on creativity and values (Taylor, 2010; Taylor & Kaufman, 2020). Participants wrote short stories to two different titles (“Frame” and “Glow”) in random order about seven days apart. Participants were given 15 minutes to complete each story. Five quasi-expert raters (advanced undergraduate or graduate students studying creativity) evaluated the stories. Any analyses without the full 163 participants was either because participants did not complete both stories or because stories were not rated. The stories from both prompts were 156.86 words in length on average (*SD* = 68.03, range = 27–405).

### Results

#### Validation with human ratings

**Fig. 10:**
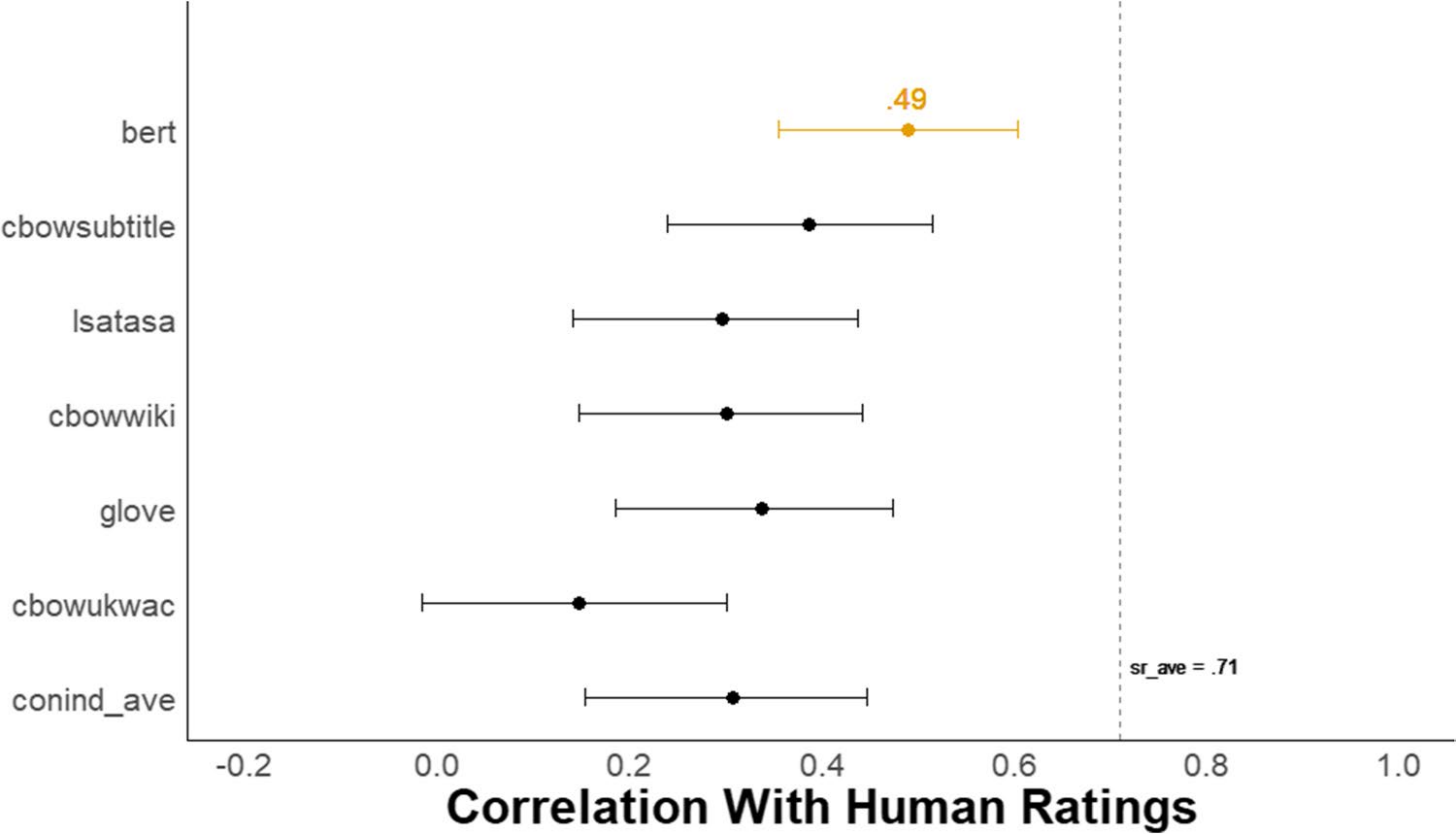
Study 4B Forest plot of correlation between all DSI models and human ratings. *N* = 147. The dots represent the correlation between each *DSI* score and the average human creativity rating with 95% CIs for the frame prompt. The dotted line, *sr_ave*, represents the average correlation between each single human rater and the average human creativity rating

Figure 10 depicts a forest plot of the Pearson correlations with 95% confidence intervals between each of the six *DSI* scores and the mean creativity scores from human raters for the frame prompt. The BERT model has a substantial advantage over all other models, with a correlation with human raters of *r* = .49, 95% CI [.35, .60]. BERT outperformed a model that averages scores from five semantic models (i.e., conind_ave) that generate context-independent word embeddings, *r* = .31, 95% CI [.15, .45] (Beaty & Johnson, 2021, conind_ave). The difference between these two correlations was reliable (difference in *r* = .18, 95% CI [.05, .31], *z* = 2.67, *p* = .008).

In addition, the BERT model correlation with human raters was lower than the average correlation for the frame prompt between each single human rater and the mean creativity rating (*sr_ave* = .71, 95% CI [.62, .78]; difference in *r* = .22, 95% CI [.07, .37], *z* = 2.98, *p* = .003).

See Supplemental Material for comparable results for the glow prompt and for evidence of incremental validity.

## Study 4C

### Method

#### Participants

In a secondary analysis of data from Zedelius et al. (2019) Study 1, participants (*N* = 133) were undergraduate students who participated for course credit (*M*_*age*_ = 19.30, *SD* = 1.40; 88 women, 44 men, 1 unidentified).

### Materials

Participants were given 20 minutes to write a story with a protagonist who had attained a superpower. The stories were 442.59 words in length on average (*SD* = 153.32, range = 71–868). There were six human raters, all trained to use a rubric to score originality, defined as the degree to which a story idea or plotline was original, that is, unlike other stories.

### Results

#### Validation with human ratings

**Fig. 11:**
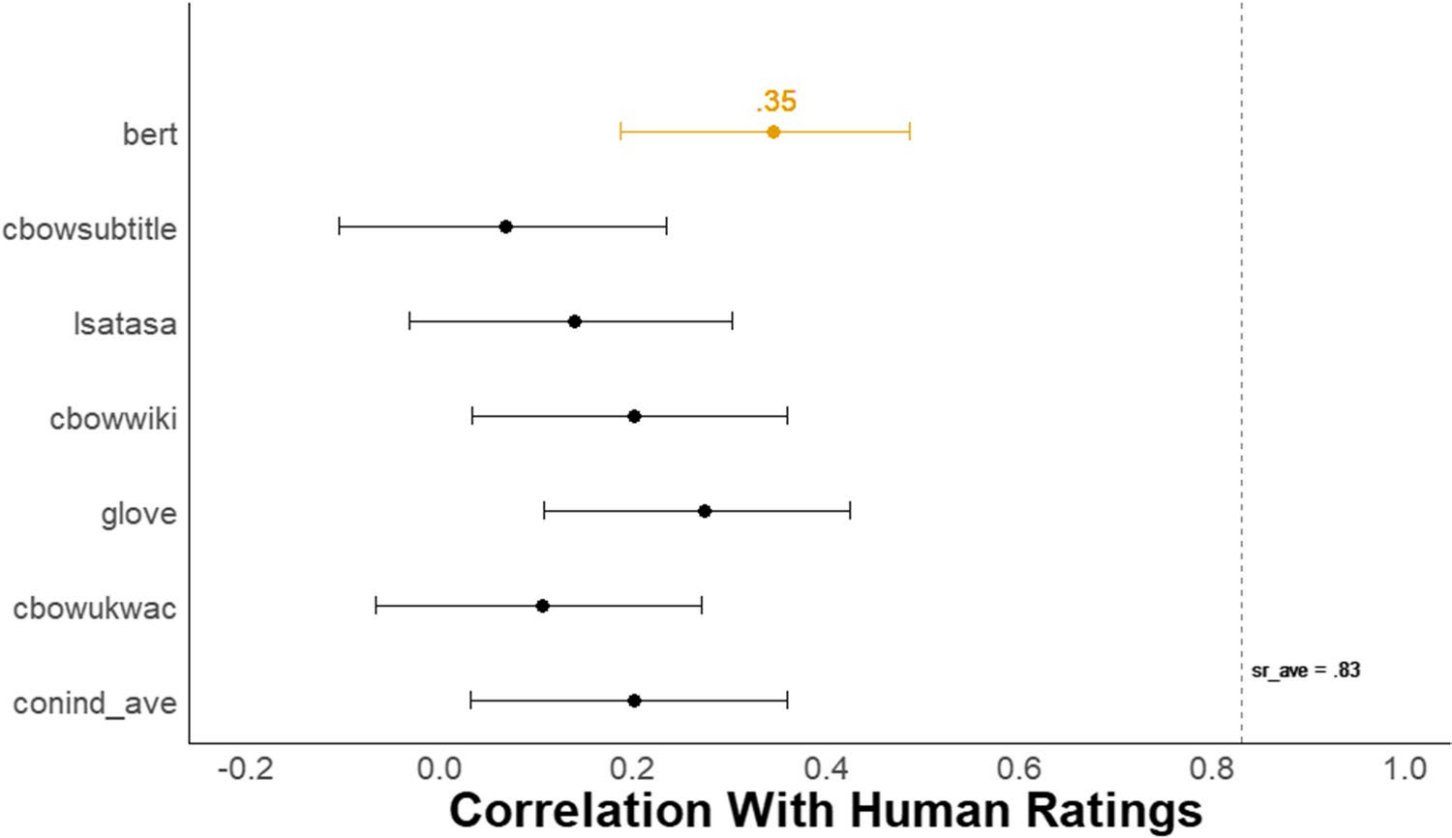
Study 4C Forest plot of correlation between all DSI models and human ratings. *N* = 133. The dots represent the correlation between each *DSI* score and the average human creativity rating with 95% CIs for the first study from Zedelius et al. (2019). The dotted line, *sr_ave*, represents the average correlation between each single human rater and the average human creativity rating

Figure 11 shows a forest plot of the Pearson correlation with 95% confidence intervals between each of the six *DSI* scores and the mean creativity scores from human raters for the first study from Zedelius et al. (2019). Again, the BERT model had a substantial advantage over all other models, with a correlation with human raters of *r* = .35, 95% CI [.19, .49]. BERT outperformed a model that averages scores from five semantic models (i.e., conind_ave) that generate context-independent word embeddings, *r* = .20, 95% CI [.03, .36]. The difference between these two correlations was reliable (difference in *r* = .15, 95% CI [.01, .29], *z* = 2.12, *p* = .034).

In addition, the BERT model correlation with human raters was substantially lower than the average correlation between each single human rater and the mean creativity rating (*sr_ave* = .83, 95% CI [.77, .88]; difference in *r* = .48, 95% CI [.33, .65], *z* = 6.63, *p* < .001).

See Supplemental Material for results for the second study from Zedelius et al. (2019).

## Study 5

The results of Studies 4A–4C showed that *DSI* exhibited a strong correlation with human creativity ratings across diverse prompts and creative story tasks. However, an examination of all studies thus far reveals that, generally, *DSI* exhibits higher correlations with human creativity ratings for shorter stories than for longer stories. This trend could stem from a number of factors. One possibility is that there is an artifact in the computational architecture, whereby higher word counts systematically impact the *DSI* metric, independently of the extent to which the story connects divergent ideas. Another possibility is that longer stories permit more sophisticated writing elements such as character development, voice, and world-building, and consequently one’s ability to connect divergent ideas is just one among many other elements raters use to judge the creativity of a story, and as a result receives less weight (see “General discussion”).

The purpose of Study 5 was to systematically investigate whether text length produces an artifact in *DSI* scores. The potential confound of text length in linguistic indices is well known (Malvern et al., 2004; McCarthy & Jarvis, 2010). For example, lexical diversity, that is, the range of unique words used in a text, can be systematically biased by text length (Malvern et al., 2004). One way to assess lexical diversity is using the type–token ratio, which simply takes the ratio between the number of unique words over the total number of words in a text (McCarthy & Jarvis, 2010). The issue is that the longer the text, the less likely the writer continues to generate unique words, so there is a predictable decreasing slope in the type–token ratio as text length increases. Ideally, automated linguistic metrics should represent the construct of interest (e.g., degree to which a story connects divergent ideas) independent of text length.

Following a well-validated approach, McCarthy and Jarvis (2010) developed a measure of lexical diversity that was independent of text length. We will implement a similar validation approach to determine whether *DSI* is systematically biased by text length. This involves generating a corpus (i.e., collection of stories) and then randomly sampling subsections of each story of varying text lengths without replacement until the entire text is sampled, computing *DSI* on each subsection, and then determining whether the metric’s central tendency, variability, or distribution changes as a function of text length (McCarthy & Jarvis, 2010). The key control is that the same corpus is used across all comparisons of text length, so there are no other factors (e.g., writing ability, word frequency, vocabulary level) that vary in the text length comparisons.

### Corpora for text length analysis

Following McCarthy and Jarvis’ (2010) validation method, we created three text corpora of short stories that were 90 words, 200 words, or 2000 words in length. The first corpus was created from the Kaufman et al. (2013) data set (current paper Study 3B), where we selected a random sample of 60 stories that were at least 90 words in length (after stop word removal), and then truncated the stories so that each story was precisely 90 words in length. The second short story corpus was created from the Zedelius et al. (2019) Study 1 data set (current paper Study 4C), where we selected a random sample of 60 stories that were at least 200 words in length (after stop word removal), and then truncated the stories so that each story was precisely 200 words in length. The third corpus was the same corpus used in many other studies validating linguistic indices (e.g., McCarthy & Jarvis, 2010), the freely available Lancaster-Oslo-Bergen (LOB) corpus, which includes stories from the following domains: narrative fiction, academic prose, journalist articles, editorials, popular lore, and biographies (Johansson et al., 1978). We randomly selected 200 stories from the LOB corpus that were each 2000 words in length.

As Table 2 shows, for each corpus, we randomly sampled, without replacement, a number of equal-sized subsections of each story, so that all words in every story were always used at each text length. For example, for the Zedelius et al. (2019) corpus and a subsection size of 50 words, we randomly selected (without replacement) four samples of 50 words to cover the entire 200-word story—and did this for each of the 60 stories. Then, *DSI* (BERT model) was computed on each subsection of 50 words for each story. Finally, those four *DSI* scores were averaged to obtain a single *DSI* score for each story.

**Table 2:** Text length and number of randomly sampled subsections for each length

Kaufman et al. (2013) corpus		Zedelius et al. (2019) corpus		LOB corpus	
Text length (no. of words)	No. of subsections	Text length (no. of words)	No. of subsections	Text length (no. of words)	No. of subsections
90	1	200	1	2000	1
45	2	100	2	1000	2
30	3	50	4	666	3
18	5	25	8	500	4
10	9	10	20	400	5
-	-	-	-	333	6
-	-	-	-	250	8
-	-	-	-	200	10
-	-	-	-	100	20

### Text length impact on *DSI*

**Fig. 12:**
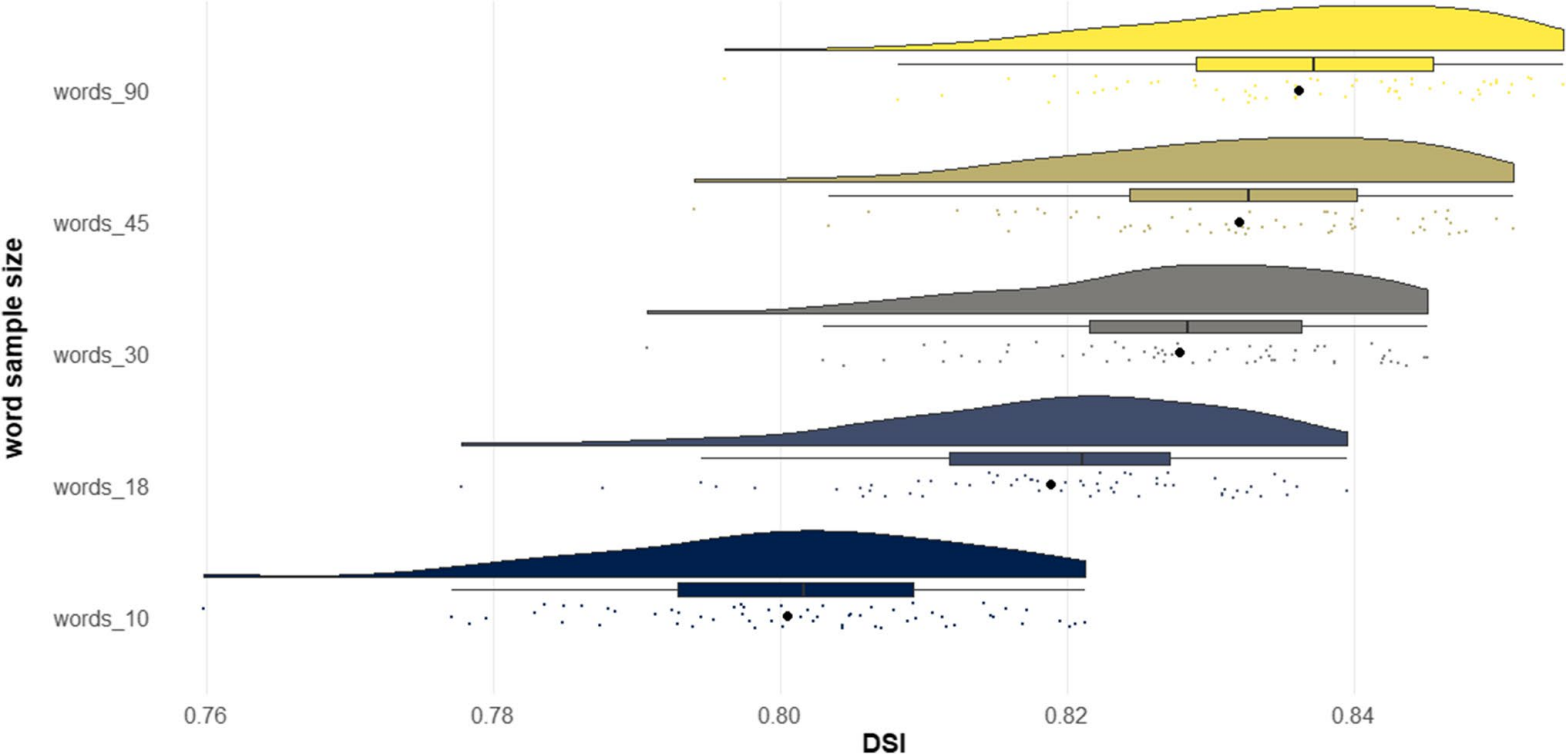
Study 5 Kaufman et al. (2013) corpus - Text length distribution comparison for BERT DSI. *N* = 60. words_10 = number of words randomly sampled without replacement until all samples were extracted from the 90-word story from each of 60 stories

**Fig. 13:**
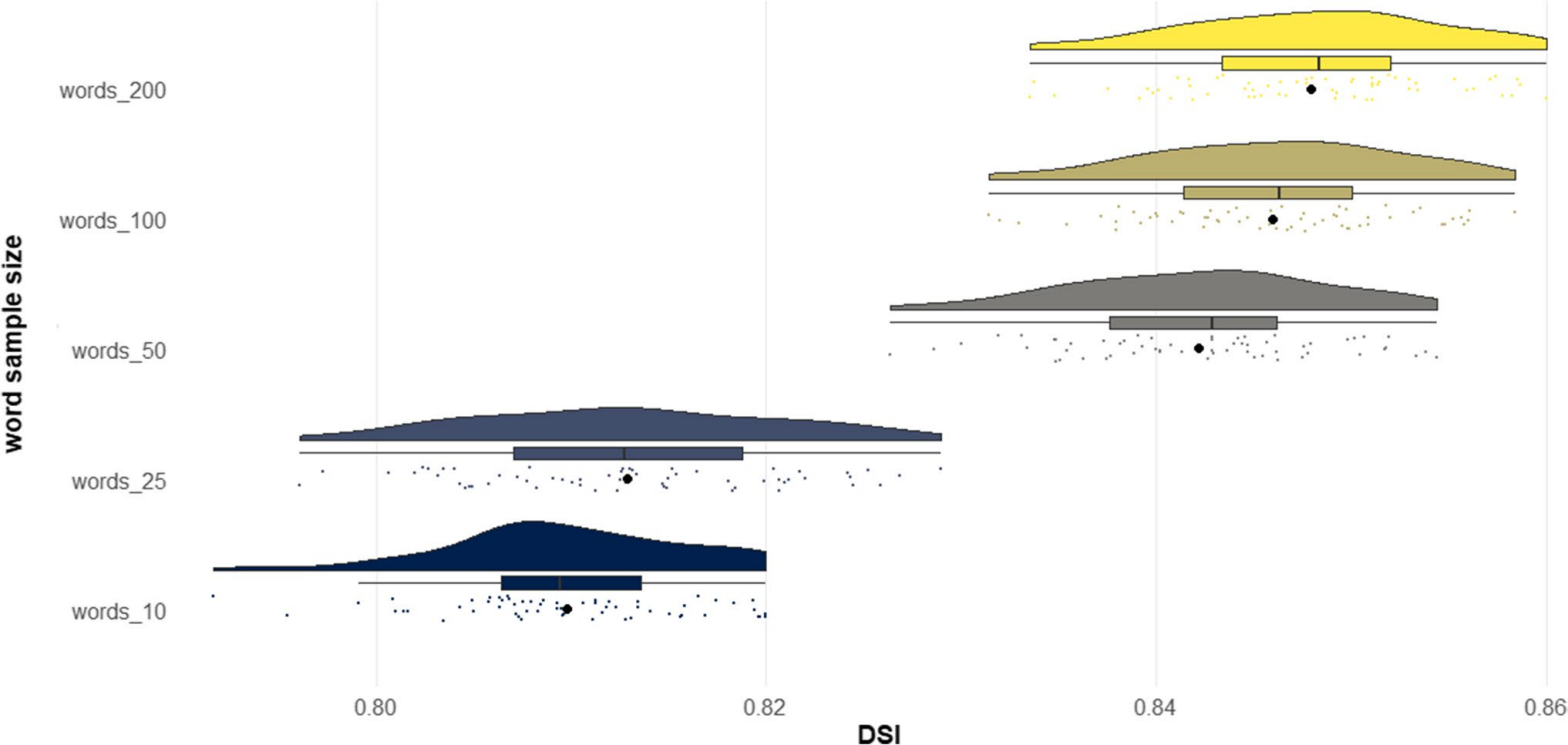
Study 5 Zedelius et al. (2019) corpus - Text length distribution comparison for BERT DSI. *N* = 60. words_10 = number of words randomly sampled without replacement until all samples were extracted from the 200-word story from each of 60 stories

**Fig. 14:**
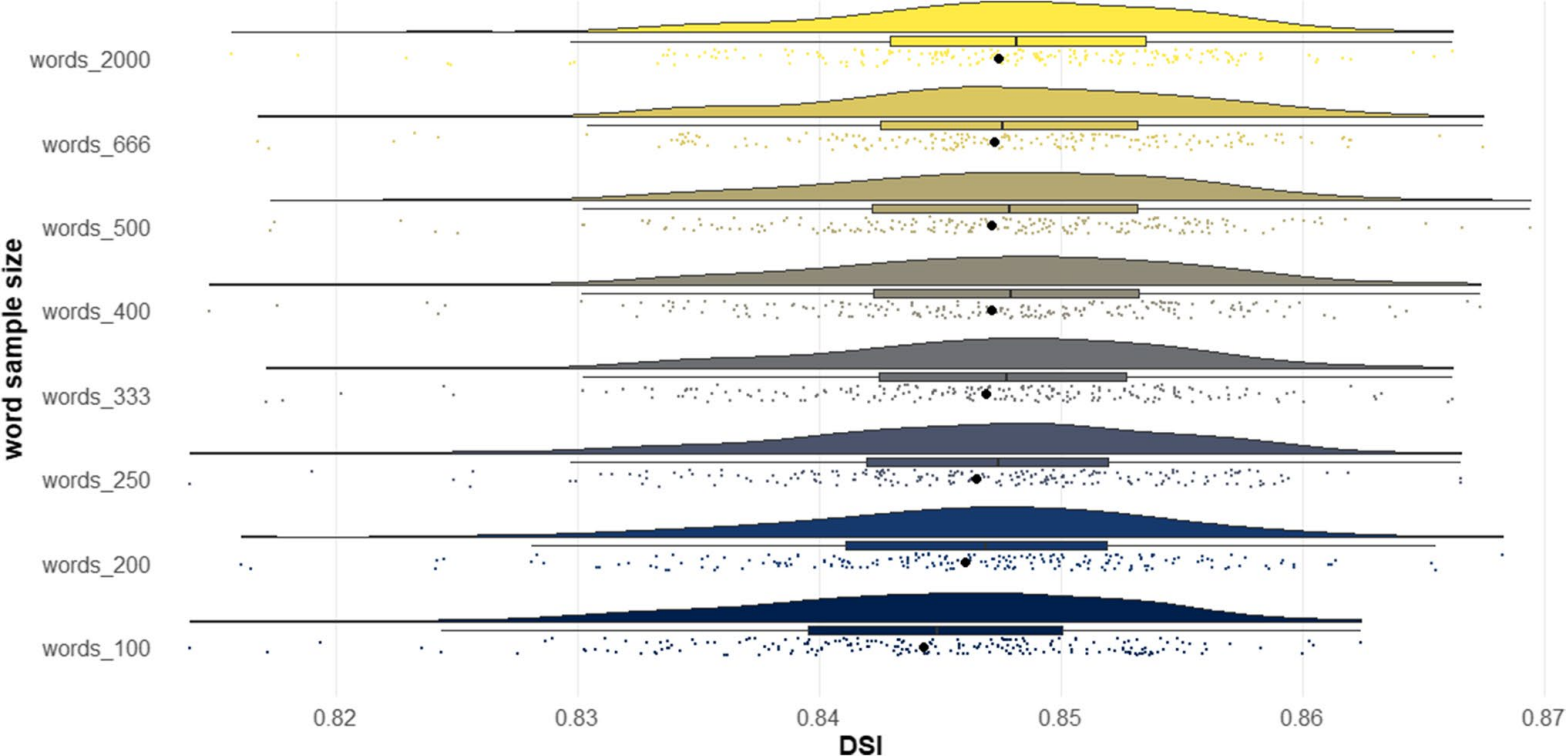
Study 5 LOB corpus - Text length distribution comparison for BERT DSI. *N* = 200. words_500 = number of words randomly sampled without replacement until all samples were extracted from the 2000-word story from each of 200 stories from the LOB corpus

As Figs. 12, 13, and 14 show for the Kaufman et al. (2013) corpus, Zedelius et al. (2019) corpus, and LOB corpus, *DSI* scores increased with text length up to text length of 30 words, 50 words, and 200 words, respectively. Then, for any additional increase in text length, the central tendency, variability, and distribution shape were all quite similar. In addition, as Tables 3, 4 and 5 show, the rank order of participants’ scores did not change substantively when the text length reached these same values, where the correlations between the 30-word, 50-word, and 200-word text length scores and the full-length story scores, respectively across corpora, were *r*s > .96.

**Table 3:** Study 5 Taylor and Kaufman (2020) - correlations between each text length with confidence intervals

Variable	*M*	*SD*	1	2	3	4
1. words_10	0.80	0.01				
2. words_18	0.82	0.01	.96**			
			[.93, .97]			
3. words_30	0.83	0.01	.95**	.98**		
			[.92, .97]	[.96, .99]		
4. words_45	0.83	0.01	.97**	.98**	.99**	
			[.94, .98]	[.97, .99]	[.98, .99]	
5. words_90	0.84	0.01	.97**	.99**	.99**	1.00**
			[.95, .98]	[.98, .99]	[.99, 1.00]	[.99, 1.00]

*M* and *SD* are used to represent mean and standard deviation, respectively. Values in square brackets indicate the 95% confidence interval for each correlation. The confidence interval is a plausible range of population correlations that could have caused the sample correlation (Cumming, 2014). * indicates *p* < .05. ** indicates *p* < .01

**Table 4:** Study 5 Zedelius et al. (2019) corpus - Correlations between each text length with confidence intervals

Variable	*M*	*SD*	1	2	3	4
1. words_10	0.81	0.01				
2. words_25	0.81	0.01	.79**			
			[.67, .87]			
3. words_50	0.84	0.01	.93**	.88**		
			[.88, .96]	[.80, .93]		
4. words_100	0.85	0.01	.94**	.88**	.99**	
			[.90, .96]	[.80, .93]	[.98, .99]	
5. words_200	0.85	0.01	.94**	.88**	.99**	1.00**
			[.90, .96]	[.81, .93]	[.99, .99]	[1.00, 1.00]

*M* and *SD* represent mean and standard deviation, respectively. Values in square brackets indicate the 95% confidence interval for each correlation. The confidence interval is a plausible range of population correlations that could have caused the sample correlation (Cumming, 2014). * indicates *p* < .05. ** indicates *p* < .01

**Table 5:** Study 5 LOB corpus - Correlations between each text length with confidence intervals

Variable	*M*	*SD*	1	2	3	4	5	6	7
1. words_100	0.84	0.01							
2. words_200	0.85	0.01	.95**						
			[.93, .96]						
3. words_250	0.85	0.01	.95**	.96**					
			[.93, .96]	[.95, .97]					
4. words_333	0.85	0.01	.94**	.96**	.97**				
			[.91, .95]	[.94, .97]	[.96, .97]				
5. words_400	0.85	0.01	.95**	.96**	.97**	.97**			
			[.93, .96]	[.95, .97]	[.96, .98]	[.96, .98]			
6. words_500	0.85	0.01	.94**	.95**	.97**	.97**	.98**		
			[.93, .96]	[.94, .97]	[.96, .98]	[.96, .98]	[.97, .98]		
7. words_666	0.85	0.01	.94**	.96**	.97**	.97**	.98**	.98**	
			[.92, .96]	[.94, .97]	[.96, .98]	[.96, .98]	[.97, .98]	[.97, .98]	
8. words_2000	0.85	0.01	.96**	.97**	.98**	.97**	.98**	.98**	.99**
			[.94, .97]	[.96, .97]	[.97, .98]	[.96, .98]	[.98, .99]	[.98, .99]	[.98, .99]

*M* and *SD* are used to represent mean and standard deviation, respectively. Values in square brackets indicate the 95% confidence interval for each correlation. The confidence interval is a plausible range of population correlations that could have caused the sample correlation (Cumming, 2014). * indicates *p* < .05. ** indicates *p* < .01

## Study 6

An analysis of text length on *DSI* revealed that scores stabilize between 30 and 50 words for stories between 90 and 200 words in length, well before the average length of stories in any of the current studies. Consequently, the lower correlations between *DSI* and human creativity ratings cannot be explained by a text length artifactual effect on *DSI* scores. See “General discussion” for alternative explanations.

The primary goal of Study 6 was to determine whether *DSI* generalizes to English language learners and different cultural groups. A critical step in establishing algorithmic fairness is to investigate potential bias in automated assessment across different cultural and language groups (Friedler et al., 2019). In addition, sociocultural context will play a role in the humans who rate creative work, the content of the writer’s work, and the identities of the creative writers (Alhusaini & Maker, 2015). Consequently, it will be important to compare both human creativity ratings and *DSI* scores across cultural and language groups to determine whether either set of scores advantage or disadvantage a particular group.

### Method

#### Participants

Using Prolific, 226 participants were recruited and compensated $6.50 for participation, with an average hourly rate of $11.26. We used prescreening questions to recruit 119 participants who identified as English language learners and Hispanic (*M*_*age*_ = 23.70, range = 18–58; 98 women, 20 men, 1 nonbinary) and 107 who identified as White and indicated that English was their primary language (*M*_*age*_ = 28.04, range = 18–77; 69 women, 33 men, 4 nonbinary, 1 genderqueer).

Socioeconomic status (SES) was computed in accordance with guidelines from the Bureau of Justice Statistics (Berzofsky et al., 2014) and US Department of Health and Human Services (2021) with a composite of educational attainment, income relative to the federal poverty level and number of people in the household, employment status, and housing status. SES was moderately higher in the L1-White group (*M* = 4.50, *SD* = 1.80) than in the L2-Hispanic group (*M* = 3.42, *SD* = 1.56), *t* = 4.82, *p* < .001, *d* = .64, 95% CI [.38, .96].

In the L2-Hispanic group, 73% reported Spanish as the first language they learned, and in the L1-white group, 84% reported English as the first language they learned. The L1-White group reported that they were exposed to English 93.81% (*SD* = 11.14%) of the time, compared with the L2-Hispanic who reported exposure to English 33.78% (*SD* = 17.41%) of the time. Finally, L1-White participants (*M* = 87.45, *SD* = 12.09) performed equivalently to L2-Hispanic participants (*M* = 87.69, *SD* = 8.63) on the LexTALE (Lexical Test for Advanced Learners of English) English vocabulary test (Lemhöfer & Broersma, 2012).

### Materials

#### Five-sentence creative story

Participants were given the five-sentence creative story task (Prabhakaran et al., 2014). They were given the three-word prompt, stamp-letter-send. The creativity of the stories was evaluated by three trained undergraduates (who were blind to participant identity) following the same procedure as Study 1. The stories were 86.37 words in length on average (*SD* = 32.48, range = 21 – 202) and participants were given five minutes to write.

#### Criterion creativity measures

##### Openness to experience

Participants completed the openness to experience subscale of the Big Five Aspects Scale (BFAS; DeYoung et al., 2007). The openness subscale of the openness/intellect scale measures a preference for aesthetics and creativity (e.g., “I believe in the importance of art”; 1 = *strongly disagree*, 5 = *strongly agree*). Reliability was adequate, with omega = .67.

##### Inventory of Creative Activities and Achievements—Writing

Participants completed the ICAA-Writing subsection (Diedrich et al., 2018), where the term “literature” was replaced with “writing,” given that the items involved writing specifically and writing was the creative activity of interest in the current study (e.g., “Wrote a short literary work”). They received activity (omega = .66) and achievement scores (summary score only).

##### Creative self-efficacy

Participants completed the creative self-efficacy (CSE) subscale of the Short Scale of Creative Self (SSCS; Karwowski, 2014). CSE measures the extent to which people see themselves as capable of solving creative challenges, such as “I am good at proposing original solutions to problems” (1 = *definitely not*, 5 = *definitely yes*). Reliability was excellent, with omega = .92.

### Results

#### Comparison of semantic models and relation to human ratings

**Fig. 15:**
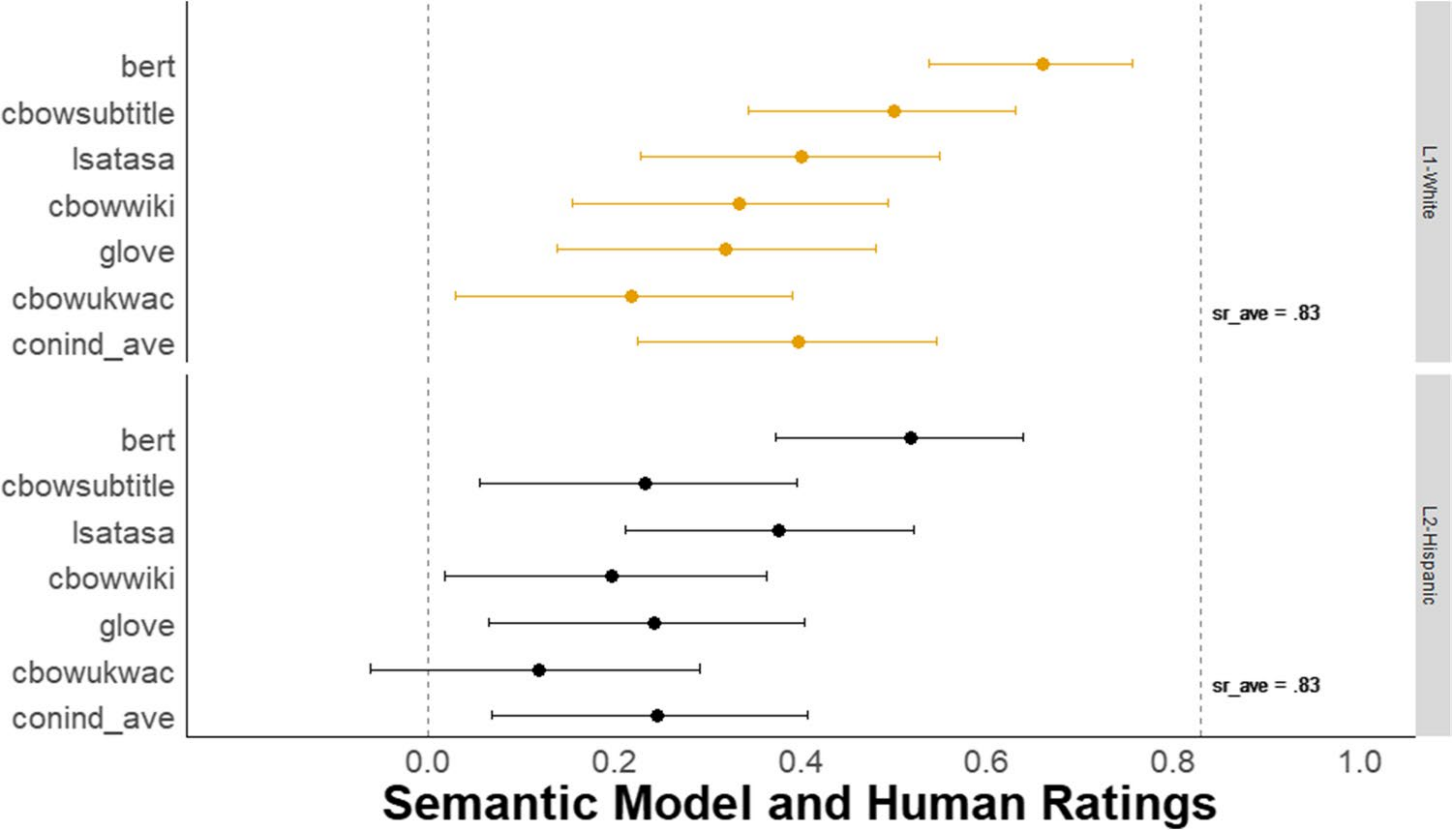
Study 6 Comparison of L1-White and L2-Hispanic on relation between semantic models and human ratings. *N* = 226. The dots represent the correlation between each *DSI* score and the average human creativity rating with 95% confidence intervals. The dotted line, *sr_ave*, represents the average correlation between each single human rater and the average human creativity rating across both groups

Figure 15 shows a forest plot of the Pearson correlation with 95% confidence intervals between each of the six *DSI* scores and the mean creativity scores from three human raters. Again, the BERT model had an advantage over all other models. The correlation between BERT *DSI* scores and human ratings was slightly higher for L1-White (*r* = .66, 95% CI [.54, .76]) compared with L2-Hispanic (*r* = .52, 95% CI [.37, .64]) participants, but the difference between these two correlations was not reliable (difference in *r* = .14, *z* = 1.60, *p =* .109, 95% CI [−.03, .31]), providing preliminary evidence that *DSI* is not significantly biased against either group.

In addition, the BERT model correlation with human raters was lower than the average correlation between each single human rater and the mean creativity rating (*sr_ave* = .85, 95% CI [.81, .88]).

#### Comparison of L1-White and L2-Hispanic groups on BERT *DSI*

There was no reliable difference in human creativity ratings between the L1-White (*M* = 2.72, *SD* = 0.90) and L2-Hispanic (*M =* 2.82, *SD =* 0.71) participants, *t*(202.52) = 0.88, *p* = .381, *d* = .12, 95% [−.39, .16]. However, L2-Hispanic participants (*M* = .803, *SD* = .013) scored slightly higher on BERT *DSI* scores than L1-White participants (*M* = .799, *SD* = .017), *t*(202) = 2.20, *p* = .029, *d* = .30, 95% CI [.02, .55].

#### Convergent and criterion-related validity

**Fig. 16:**
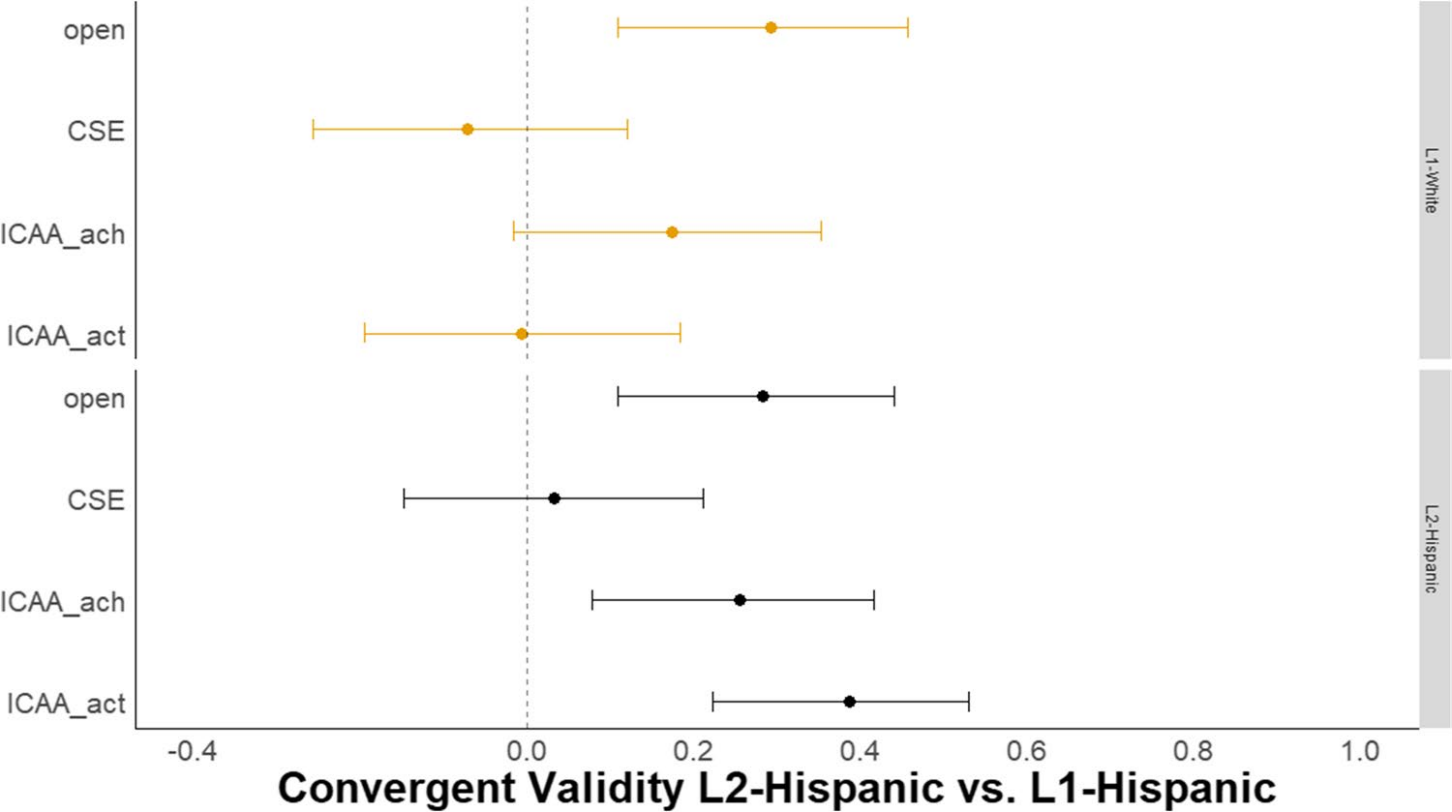
Study 6 Convergent and criterion-related validity of BERT DSI across L1-White and L2-Hispanic groups. *N =* 226. The dots represent the correlation between each convergent and criterion-related creativity measure and BERT *DSI* scores with 95% confidence intervals. open = openness to experience; CSE = creative self-efficacy; ICAA-ach = Inventory of Creative Activities and Achievement writing achievement score; ICC_act = Inventory of Creative Activities and Achievement writing activities score

Criterion-related validity for openness to experience replicated Study 1 findings across L1-White and L2-Hispanic participants (Fig. 16). There is weak evidence for the validity for creative activities and achievement in writing for L2-Hispanic participants, but not for L1-White participants. Thus, although the BERT model was trained on English texts, *DSI* values derived from L2-Hispanic individuals correlated more highly with external creativity measures than L1-White individuals.

#### Summary table

Table 6 provides a summary of critical information across all studies. The table shows that there is substantial variability in the correlation between BERT *DSI* and human creativity ratings across creativity prompts, with validity coefficients in a range of *r* = .09–.77. Consequently, researchers should select the creative story task and prompt carefully when using BERT *DSI* as a strong predictor of creativity. The table provides context for this variability including word count and how well the average human rater correlated with the human creativity rating mean. While there does appear to be a trend for higher word counts leading to lower validity coefficients, there does not appear to be a strong relationship between validity coefficients and *sr_ave*. However, neither story length nor inter-rater reliability was systematically manipulated across studies (see Study 5 for systematic investigation of story length), so conclusions should be drawn with caution.

**Table 6:** Summary table for all studies ordered by correlation between BERT DSI and human raters

Study no.	Secondary source	Prompt	Word count *M* (*SD*)	Sample size	sr_ave [95% CI]	*r* [95% CI]
1	-	stamp-letter-send	59.75 (20.17)	179	.89 [.85, .92]	.77 [.70, .82]
3A	T	p1 - 3 images	52.11 (43.63)	125	.86 [.80, .90]	.73 [.62, .81]
6	-	stamp-letter-send	80.36 (30.68)	107 L1	.86 [.80, .90]	.66 [.54, .76]
2	-	stamp-letter-send	73.79 (23.35)	153	.84 [.80, .88]	.61 [.50, .70]
2	-	petrol-diesel-pump	73.22 (23.66)	153	.87 [.82, .90]	.59 [.47, .68]
4A	W	shade (cool or sunny)	39.79 (22.93)	86	.77 [.66, .84]	.59 [.43, .71]
2	-	year-week-embark	68.05 (20.39)	153	.85 [.80, .89]	.57 [.45, .67]
4A	W	marriage (satisfying or divorced)	38.67 (19.05)	86	.74 [.63, .83]	.56 [.40, .69]
2	-	statement-stealth-detect	68.30 (22.61)	153	.79 [.73, .85]	.55 [.43, .65]
3A	T	p4 - 3 images	64.95 (62.59)	125	.82 [.75, .87]	.55 [.40, .67]
4A	W	joy (cheerful or painful)	42.09 (20.96)	86	.70 [.58, .80]	.55 [.38, .68]
2	-	belief-faith-sing	67.39 (21.29)	153	.84 [.78, .88]	.54 [.41, .64]
4A	W	sky (blue or grounded)	41.53 (19.61)	86	.71 [.59, .80]	.54 [.37, .68]
4A	W	death (tragic or living)	44.72 [25.12)	86	.69 [.57, .79]	.54 [.37, .68]
2	-	organ-empire-comply	68.33 (20.73)	153	.80 [.73, .85]	.52 [.40, .63]
4B	TK	glow	155.50 (69.33)	141	.76 [.68, .82]	.52 [.39, .63]
6	-	stamp-letter-send	91.77 (33.22)	119 L2	.84 [.78, .88]	.52 [.37, .64]
4A	W	delay (long or speedy)	43.74 (22.18)	86	.69 [.56, .79]	.52 [.35, .66]
3B	K	execution/2305	194.23 (172.26)	202-EnglMa	.67 [.59, .74]	.50 [.39, .60]
4B	TK	frame	158.17 (66.97)	147	.71 [.62, .78]	.49 [.35, .60]
4A	W	simplicity (clear or complex)	40.46 (18.08)	86	.70 [.57, .79]	.49 [.31, .63]
3B	K	execution/2305	194.23 (172.26)	202-EducMa	.67 [.58, .74]	.48 [.36, .58]
3A	T	p3 - 3 images	61.36 (54.17)	125	.83 [.77, .88]	.48 [.32, .61]
2	-	gloom-payment-exist	68.97 (18.86)	153	.82 [.76, .87]	.47 [.34, .59]
3B	K	execution/2305	194.23 (172.26)	202-Novice	.37 [.25, .48]	.46 [.36, .56]
3B	K	execution/2305	194.23 (172.26)	202-EnglTe	.74 [.67, .80]	.46 [.34, .56]
3B	K	execution/2305	194.23 (172.26)	202-CreativStu	.73 [.65, .79]	.46 [.34, .56]
3B	K	execution/2305	194.23 (172.26)	202-Expert	.78 [.72, .83]	.43 [.31, .54]
4A	W	illness (harmful or healthy)	41.35 (20.28)	86	.72 [.61, .81]	.43 [.24, .59]
4A	W	enemy (hostile or friendly)	44.73 (20.85)	86	.72 [.60, .81]	.39 [.20, .56]
3A	T	p2 - 3 images	52.31 (44.87)	125	.87 [.82, .91]	.37 [.18, .53]
4A	W	lie (unacceptable or truthful)	44.88 (21.78)	86	.71 [.59, .80]	.36 [.16, .53]
4C	Z1	character with superpower	442.59 (153.32)	133	.83 [.77, .88]	.35 [.19, .49]
4C	Z2	character with superpower	476.17 (158.48)	128	.77 [.66, .84]	.09 [−.09, .25]

- = new data collected for this paper; T = Taylor et al. (2021); W = Ward et al. (2013); TK = Taylor & Kaufman (2020); K = Kaufman et al. (2013); Z1 = Zedelius et al. (2019) Study 1; Z2 = Zedelius et al. (2019) Study 2

## General discussion

By integrating creativity and distributional semantics theory, we developed a novel conceptualization of creativity in writing—*divergent semantic integration* (*DSI*). *DSI* is defined as the extent to which a narrative connects divergent ideas. Study 1 demonstrated a substantial advantage of using BERT (Devlin et al., 2019) to generate *DSI* scores to capture human creativity ratings and established its convergent validity, criterion-related validity, and incremental validity. This is most likely because, unlike the other models, BERT’s word embeddings are context-dependent and reflect the nuances of syntax and word choice used in narratives. For some of the studies (Study 1, 3A, 4A), the correlation between *DSI* and human creativity ratings approached human inter-rater reliability, which highlights the critical role *DSI* played in human assessments of creativity. Study 2 employed a confirmatory factor analytic approach to examine *DSI*’s ability to capture human ratings of creativity while minimizing the role of item- and rater-specific variance, and demonstrated a high correlation between *DSI* and human creativity ratings (*r* = .85), explaining over 72% of the variance. Studies 2, 3A, and 4A demonstrated that *DSI* had excellent reliability (omegas = .88, .75, and .88) and that it generalized to various creative story prompts. Studies 3A and 3B showed that *DSI* correlated highly with expert and novice ratings of creativity. Study 5 systematically investigated the influence of text length on *DSI* scores and demonstrated an artifactual effect of text length on *DSI* scores until stories were 30–50 words in length. In Study 6, evidence was provided for the generalizability of *DSI* across varying language and cultural backgrounds by showing comparable validity correlations and scores for participants who identified as Hispanic and L2 English-speakers and participants identifying as White and L1 English-speakers.

The results have potentially far-reaching and consequential implications for quantifying creativity in narrative text. Narrative text is ubiquitous in society, from job applications to journalistic stories to fictional stories. These results highlight how this integration leads to novel hypotheses about creativity and novel ways to quantify its underlying processes and components. Creativity is among the most valuable twenty-first-century skills, so developing robust tools for creativity assessment is a top priority (Florida, 2014; Lichtenberg et al., 2008). In addition, the labor cost and subjectivity of human creativity assessment impede the progress in scientific understanding of creativity as well as applications in education and industry settings. We circumvented the limitations of human creativity assessment by automating the assessment of *DSI*. These results demonstrate impressive predictive power of *DSI* in capturing creativity in narrative text. As previous work shows minimal predictive power for automated assessments of creativity in narrative text, these results represent a substantial step forward for researchers from diverse disciplines including psychology (D’Souza, 2021; Toubia et al., 2021; Zedelius et al., 2019), linguistics (Mozaffari, 2013), education (Graham et al., 2002; Vaezi & Rezaei, 2019), and creative writing (Bland, 2011).

### Theoretical implications and new directions

Distributional semantic modeling is undergoing an exponential expansion of models which will spark completely new hypotheses about creativity components and processes. For example, multi-modal semantic models that integrate more than just text information, such as visual images and other multisensory information, are taking shape (Bisk et al., 2020; Kiela et al., 2016; McClelland et al., 2019; Ruis et al., 2020). There is an active debate in the creativity literature about how the presentation of images versus text during the idea generation process influences top-down versus bottom-up processing (Chrysikou et al., 2016). Multi-modal distributional semantic models that incorporate visual and text input could help resolve these debates and permit new designs, new questions, and new operationalizations. In addition, rapid progress is being made on how to go from word embeddings to idea and concept embeddings, critical if a semantic model is to represent human cognition (Eisenstein, 2019; Lake & Murphy, 2021). Relatedly, a limitation of the current operationalization of *DSI* is that it is designed to capture the degree to which a story connects divergent *ideas*, yet it is rooted in the semantic distance between words, not ideas.

The integration of creativity theory and distributional semantics theory highlights the potential to generate novel conceptualizations and quantifications of creativity and its underlying components. For example, *DSI* provides a means to map cognitive mechanisms of complex and naturalistic creative behaviors, such as creative writing. Here, we have shown that the ability to connect remotely associated ideas in a story—that is, *DSI*—correlates with individual differences in cognitive abilities, including fluid intelligence (Gf), crystallized intelligence (Gc), and broad retrieval ability (Gr; cf. Taylor & Barbot, 2021). These findings are consistent with prior work on creativity that highlight the importance of general cognitive abilities (Frith et al., 2021; Gerwig et al., 2021; Nusbaum & Silvia, 2011; Sternberg, 2006; Stevenson et al., 2021). In the context of creative writing, our findings indicate that a person’s ability to craft a short story may rely in part on the breadth of their general knowledge (Gc). Moreover, given the role of Gf in convergent thinking, Gf may play a role in the integrative aspect of story writing, where a writer weaves concepts together into a cohesive and compelling narrative. This interpretation remains speculative, however, and we await future research to take a more granular approach to studying the cognitive mechanisms underlying writing ability, leveraging the power of *DSI* to ask specific questions regarding the role of Gf/convergent thinking in writing ability (e.g., via experimental manipulations).

Importantly, studying the role of intelligence in creativity is merely one of many potential lines of research applications with *DSI*. Given the richness and pervasiveness of narrative text, and its potential to explore creative processes in diverse contexts outside of the lab, we see many opportunities for future research. For example, *DSI* could be used to longitudinally study how narrative complexity tracks with daily fluctuations in affective processes relevant for creativity, within a single individual over multiple timescales (e.g., combining *DSI* with momentary experience sampling of emotional experiences in daily life). In this case, *DSI* could be used to test theories on the role of positive emotion in creative thinking/writing, providing a computational means to link fluctuations in emotion to narrative complexity in daily life. Indeed, researchers have already begun to apply distributional semantic models to study creativity outside the lab, such as Gray et al. (2019), who found that expert-level creative achievement correlated with measures of forward flow applied to Twitter posts, supporting the theory that free association ability characterizes highly creative individuals. These are only a few examples of real-world applications of distributional semantic models in creativity research, and its application is constrained only by the imagination of researchers—and of course, by the psychometric properties of *DSI*, which must be established in new contexts and domains.

These findings contribute to the rapidly expanding application of automated language analysis in domains such as attitudes and emotions (Caluori et al., 2020; Eichstaedt et al., 2021), cultural similarities and differences (Jackson et al., 2021), and creativity (Beaty & Johnson, 2021; Dumas et al., 2020; Gray et al., 2019; Green, 2016; Heinen & Johnson, 2018; Johnson et al., 2021; Prabhakaran et al., 2014). Most prior work in validating the use of automating language analysis for creativity assessment has focused on word association tasks and the alternative uses task of divergent thinking (Beaty & Johnson, 2021; Dumas et al., 2020; Gray et al., 2019; Prabhakaran et al., 2014). However, the automated creativity assessments in prior work that focused on narrative text did not demonstrate strong capacity to predict human-rated creativity (Zedelius et al., 2019). In addition, our *DSI* metric is among the first automated creativity metrics to focus on idea diversity (see Johnson et al., 2021, for prior work with a word association task), and is the first to demonstrate consistent evidence of validity across a range of writing prompts and external creativity measures.

The current studies represent one of the first applications of context-dependent word embeddings (i.e., from BERT) in the psychological literature (Eichstaedt et al., 2021). Prior work shows that these models substantially outperform context-independent word embedding models (e.g., word2vev, Mikolov et al., 2013; GloVe, Pennington et al., 2014) in tasks like named entity recognition and question answering (BERT, Devlin et al., 2019; RobBERTa, Liu et al., 2019). Consistent with the prior literature, the current paper shows that context-dependent word embeddings, in nearly all data sets, substantially outperformed models that generate context-independent embeddings in capturing creativity in narrative text.

### Algorithmic fairness and bias

Consideration of the sociocultural context in the automated assessment of creativity is critical to establishing “algorithmic-fairness.” Prioritizing the examination of similarities and differences between varying cultural and language backgrounds is paramount to “upending racism in psychological science” (Buchanan et al., 2021). Any algorithm developed has the potential to reflect the same biases present in society (Friedler et al., 2019; Garg et al., 2018; Kiritchenko, & Mohammad, 2018; O’Neil, 2016; Venkatasubramanian & Alfano, 2020). The primary goal of Study 6 was to investigate potential similarities and differences between individuals identifying as Hispanic and L2 English speakers and individuals identifying as White and L1 English speakers. We found evidence of both similarities (strong predictive power) and differences, where convergent and criterion-related validity was slightly stronger for Hispanic L2 individuals. In addition, individuals identifying as Hispanic and L2 scored slightly higher on *DSI*, but not when using human creativity ratings, in comparison with L1 White individuals. This represents only a preliminary step to investigating the sociocultural context in the realm of automated creativity assessment. Future studies should examine many other sociocultural and language groups.

Transparency and replicability are paramount for scientific progress and promoting fairness in assessment (Friedler et al., 2019; Nosek et al., 2015). These goals can be difficult to achieve with human raters, as the criteria they use to determine what constitutes a creative response are often not reported and sometimes deliberately not discussed (i.e., the CAT). In addition, each set of human ratings of the same data will likely generate different scores due to subjective interpretation of criteria, rater fatigue, and variability in rater leniency (D’Souza, 2021; Forthmann et al., 2017). By contrast, transparency and replicability are readily achievable with algorithmic-based assessment when code and data are openly available.

### Creativity assessment implications and limitations

It is worth reiterating that *DSI* is meant to capture just one key component of creativity in writing—that is, the extent to which a story connects divergent ideas. There are many other dimensions that contribute to creativity in writing, including imagery, voice, character development, and word-building (D’Souza, 2021; Zedelius et al., 2019). This may be relevant to the general trend across the current studies that showed lower correlations between *DSI* and human creativity ratings with longer stories. A systematic analysis of text length in Study 5 showed that *DSI* scores increased with text length until random subsections of a story reached 30–50 words in length (within 90-word and 200-word stories, respectively). This finding argues strongly against a text length computational artifact as the explanation for lower correlations between *DSI* and human creativity ratings, as scores stabilized well before text lengths approached the average of the longest stories used in the current studies (i.e., Zedelius et al., 2019, ~500 words on average). In fact, when much longer stories were investigated (i.e., LOB corpus, 2000 words), the *DSI* scores stabilized when the subsection size was only 10% of the total word count (i.e., 200 out of 2000 total words). To speculate, it could be that as stories get longer, there is more opportunity for writers to incorporate other creative text elements like those mentioned above. Consequently, humans weigh more criteria when judging the overall creativity of a story (e.g., voice and imagery in Zedelius et al., 2019, current Study 4C), diminishing the relative weighting of the extent to which the story connected divergent ideas. It will be important for future studies to investigate how to capture other characteristics of creativity in writing. For example, Toubia et al. (2021) used distributional semantic modeling to capture how quickly a story moves through semantic space and how often a story returns to the same areas of semantic space.

We have emphasized the disadvantages of human creativity ratings (i.e., labor-intensiveness, subjectivity, lack of transparency); however, it will be important for future research to determine when automated versus human ratings will be best applied. An advantage of using expert human raters with domain-specific knowledge is that they are more likely to recognize common, cliché, or plagiarized products (Kaufman & Baer, 2012). However, others argue that overreliance on the CAT continues to cause a legitimation crisis in the field of creativity (Barbot et al., 2019). In addition, some have argued that experts are prone to inflexibility and consequently stifle creativity (Frensch & Sternberg, 1989). However, other research showed that although moderate expertise was associated with inflexibility, high levels of expertise was associated with the greatest flexibility (Bilalić et al., 2008).

Much work is needed before *DSI* can be applied to any high-stakes evaluation in real-world settings (e.g., Friedler et al., 2019). For example, it seems unlikely that the current *DSI* algorithm could distinguish between highly creative texts and a collection of nonsense random words. If a random word generator produced a collection of words, they would likely come from quite disparate contexts and consequently would exhibit a high *DSI* score, despite not making any sense. It will be important for future work to develop automated metrics to detect nonsense responses.

## Conclusion

The current studies demonstrated that *DSI* is a critical component of creativity in narratives. Across 3500 narratives, *DSI* demonstrated impressive predictive power of human creativity ratings and criterion measures of creativity. The findings highlight how the integration of creativity theory and distributional semantics theory has substantial potential to generate novel hypotheses about creativity and to quantify its underlying processes and components. To facilitate new discoveries across diverse disciplines, we provide access to a tutorial with code (osf.io/ath2s) on how to compute *DSI*, and have incorporated this code into an online web app (osf.io/ath2s) that allows users to upload narratives and automatically retrieve *DSI* scores.

## Data Availability

An earlier version of this paper is available on PsyArXiv at 10.31234/osf.io/fmwgy and all materials, code, and data were made available on December 21^st^, 2021 on OSF (https://osf.io/ath2s/). These studies were not preregistered.

## Footnotes

1 Creativity is a broad and complex construct, and it can be studied in many different ways (e.g., domain-general vs. domain-specific creativity) and at many different levels (e.g., little-c vs. Big C creativity). Here, we focus on psychometric or “domain-general” creativity that can be captured by performance on verbal tasks.
